# Supplementary material for: Efficient and robust estimation of many-qubit Hamiltonians
Source: Nat Commun. 2024 Jan 8;15:311. doi: 10.1038/s41467-023-44012-5 (PMC10774346; doi:10.1038/s41467-023-44012-5)
Supplement: Supplementary file 1 — Supplementary Information [file 41467_2023_44012_MOESM1_ESM.pdf]

Supplemental Material to Efficient and robust estimation of many-qubit Hamiltonians

Daniel Stilck França,[1,2,*] Liubov A. Markovich,[3,4] V. V. Dobrovitski,[3] Albert H. Werner,[1,5] and Johannes Borregaard[3,6]

[1]*QMATH, Department of Mathematical Sciences, University of Copenhagen, Universitetsparken 5, 2100 Copenhagen, Denmark*

[2]*Univ Lyon, ENS Lyon, UCBL, CNRS, Inria, LIP, F-69342, Lyon Cedex 07, France*

[3]*QuTech and Kavli Institute of Nanoscience, Delft University of Technology, 2628 CJ, Delft, The Netherlands*

[4]*Instituut-Lorentz, Universiteit Leiden, P.O. Box 9506, 2300 RA Leiden, The Netherlands*

[5]*NBIA, Niels Bohr Institute, University of Copenhagen, Blegdamsvej 17, 2100 Copenhagen, Denmark*

[6]*Department of Physics, Harvard University, Cambridge, Massachusetts 02138, USA*

Supplementary Note 1: Selecting States and Observables to Isolate Parameters

Any Hamiltonian can be written as

$$\begin{aligned} H &= \sum_{m_1}\sum_{\alpha_1} a_{\alpha_1}^{(m_1)}\sigma_{\alpha_1}^{(m_1)} + \sum_{m_1,m_2}\sum_{\alpha_1\alpha_2} a_{\alpha_1\alpha_2}^{(m_1,m_2)}\sigma_{\alpha_1}^{(m_1)}\sigma_{\alpha_2}^{(n)} + \dots \\ &\equiv \sum_{m_1}\sum_{\alpha_1} a_{\alpha_1}^{(m_1)}\mathcal{H}_{\alpha_1}^{(m_1)} + \sum_{m_1,m_2}\sum_{\alpha_1\alpha_2} a_{\alpha_1\alpha_2}^{(m_1,m_2)}\mathcal{H}_{\alpha_1\alpha_2}^{(m_1,m_2)} + \dots, \end{aligned} \tag{1}$$

where Roman indices identify the subspace on which the operator acts, and Greek indices identify the Pauli operator, e.g. $\alpha = x$. No assumption about the dimension or structure of the hermitian Hamiltonian is needed for this expansion to be valid. For a Markovian noise environment, the evolution of a quantum system ρ_0 is described by a Master equation of the form

$$\frac{d\rho_t}{dt}\Big|_{t=0} = -i[H,\rho_0] + \sum_{m=1}^{n}\sum_{\mu,\nu=1}^{3} L_{\mu,\nu}^{(m)}(\sigma_\mu^{(m)}\rho_0\sigma_\nu^{(m)\dagger} - \frac{1}{2}\{\sigma_\nu^{(m)\dagger}\sigma_\mu^{(m)},\rho_0\}), \tag{2}$$

where H is the Hamiltonian describing the evolution of the system, $L_{\mu,\nu}^{(m)}$ are the elements of the Lindblad matrix, expressed in an operator basis consisting of the different combinations of single-qubit Pauli matrices $\{\sigma\}$. Multiplying it from the right hand side on an observable O and taking the trace, we can write

$$\begin{aligned} \frac{d}{dt}\operatorname{tr}[\rho_t O]\,|_{t=0} &= -i\sum_{m_1}\sum_{\alpha_1} a_{\alpha_1}^{(m_1)}\operatorname{tr}\left[[H_{\alpha_1}^{(m_1)},\rho_0]O\right] - i\sum_{m_1m_2}\sum_{\alpha_1\alpha_2} a_{\alpha_1\alpha_2}^{(m_1m_2)}\operatorname{tr}\left[[H_{\alpha_1\alpha_2}^{(m_1m_2)},\rho_0]O\right] - \dots \\ &\dots -i\sum_{m_1\dots m_k}\sum_{\alpha_1\dots\alpha_k} a_{\alpha_1\dots\alpha_k}^{(m_1\dots m_k)}\operatorname{tr}\left[[H_{\alpha_1\dots\alpha_k}^{(m_1\dots m_k)},\rho_0]O\right]\dots \\ &+ \sum_{m}\sum_{\mu,\nu} L_{\mu\nu}^{(m)}\operatorname{tr}\left[(\sigma_\mu^{(m)}\rho_0\sigma_\nu^{(m)\dagger} - \frac{1}{2}\{\sigma_\nu^{(m)\dagger}\sigma_\mu^{(m)},\rho_0\})O\right]. \end{aligned} \tag{3}$$

Let us introduce the notation

$$B_{\alpha_1,\dots,\alpha_k}^{(m_1,\dots,m_k)}(\rho_0,O) \equiv -i\operatorname{tr}\left[[H_{\alpha_1\dots\alpha_k}^{(m_1\dots m_k)},\rho_0]O\right]. \tag{4}$$

To isolate the coefficients $\{a_{\alpha_1}^{(m_1)}, a_{\alpha_1\alpha_2}^{(m_1m_2)},\dots\}$ we observe the n qubit state with the following density matrix

$$\rho_{\tau_i,\tau_j}^{(i,j)} = \rho_{\tau_i}^{(i)}\otimes\rho_{\tau_j}^{(j)}\otimes\rho_{n-2}, \quad \tau = \{1,2,3\} \tag{5}$$

* daniel.stilck_franca@ens-lyon.fr

where the i and j are the Pauli qubits, namely $\rho^{(i,j)}_{\tau_{i,j}} = (I + \sigma^{(i,j)}_{\tau_{i,j}})/2$, and ρ_{n-2} is the density matrix of all other qubits, which we set to be maximally mixed. Using $\sigma_\alpha\sigma_\beta = \delta_{\alpha\beta}I + i\varepsilon_{\alpha\beta\gamma}\sigma_\gamma$, we can find the following relations

$$B^{(i)}_{\alpha_1}(\rho^{(i,j)}_{\tau_i,\tau_j}, O) = \frac{1}{2}\Big(\varepsilon_{\alpha_1\tau_i\gamma}\Big(\mathrm{tr}\Big[(\sigma^{(i)}_\gamma \otimes I)O\Big] + \mathrm{tr}\Big[(\sigma^{(i)}_\gamma \otimes \sigma^{(j)}_{\tau_j})O\Big]\Big)\Big), \tag{6}$$

$$B^{(j)}_{\alpha_1}(\rho^{(i,j)}_{\tau_i,\tau_j}, O) = \frac{1}{2}\Big(\varepsilon_{\alpha_1\tau_j\zeta}\Big(\mathrm{tr}\Big[(I \otimes \sigma^{(j)}_\zeta)O\Big] + \mathrm{tr}\Big[(\sigma^{(i)}_{\tau_i} \otimes \sigma^{(j)}_{\zeta})O\Big]\Big)\Big), \tag{7}$$

$$\begin{aligned} B^{(ij)}_{\alpha_1\alpha_2}(\rho^{(i,j)}_{\tau_i,\tau_j}, O) &= \frac{1}{2}\Big(\varepsilon_{\alpha_1\tau_i\gamma}\Big(\delta_{\alpha_2\tau_j}\,\mathrm{tr}\Big[(\sigma^{(i)}_\gamma \otimes I)O\Big] + \mathrm{tr}\Big[(\sigma^{(i)}_\gamma \otimes \sigma^{(j)}_{\alpha_2})O\Big]\Big) \\ &+ \varepsilon_{\alpha_2\tau_j\eta}\Big(\delta_{\alpha_1\tau_i}\,\mathrm{tr}\Big[(I \otimes \sigma^{(j)}_\eta)O\Big] + \mathrm{tr}\Big[(\sigma^{(i)}_{\alpha_1} \otimes \sigma^{(j)}_{\eta})O\Big]\Big)\Big), \end{aligned} \tag{8}$$

where O is acting on (i,j) qubits. Selecting $O = \sigma^{(i)}_{\xi_i} \otimes \sigma^{(j)}_{\xi_j}$, we can rewrite the latter matrix element as follows

$$\begin{aligned} B^{(i)}_{\alpha_1}(\rho^{(i,j)}_{\tau_i,\tau_j}, \sigma^{(i)}_{\xi_i} \otimes \sigma^{(j)}_{\xi_j}) &= 2\varepsilon_{\alpha_1\tau_i\gamma}\delta_{\xi_i\gamma}\delta_{\xi_j\tau_j} = \begin{cases} 2\varepsilon_{\alpha_1\tau_i\gamma}\delta_{\eta\tau_j} & \text{if } \xi_i = \gamma,\ \xi_j = \eta, \\ 2\varepsilon_{\alpha_1\tau_i\gamma} & \text{if } \xi_i = \gamma,\ \xi_j = \eta,\ \eta = \tau_j, \\ 0 & \text{else} \end{cases}, \\ B^{(j)}_{\alpha_1}(\rho^{(i,j)}_{\tau_i,\tau_j}, \sigma^{(i)}_{\xi_i} \otimes \sigma^{(j)}_{\xi_j}) &= 2\varepsilon_{\alpha_1\tau_j\zeta}\delta_{\xi_j\zeta}\delta_{\xi_i\tau_i} = \begin{cases} 2\varepsilon_{\alpha_1\tau_j\zeta}\delta_{\kappa\tau_i} & \text{if } \xi_j = \zeta,\ \xi_i = \kappa, \\ 2\varepsilon_{\alpha_1\tau_j\zeta} & \text{if } \xi_j = \zeta,\ \xi_i = \kappa,\ \kappa = \tau_i, \\ 0 & \text{else} \end{cases}, \\ B^{(ij)}_{\alpha_1\alpha_2}(\rho^{(i,j)}_{\tau_i,\tau_j}, \sigma^{(i)}_{\xi_i} \otimes \sigma^{(j)}_{\xi_j}) &= 2\left(\varepsilon_{\alpha_1\tau_i\gamma}\delta_{\xi_i\gamma}\delta_{\xi_j\alpha_2} + \varepsilon_{\alpha_2\tau_j\eta}\delta_{\xi_i\alpha_1}\delta_{\xi_j\eta}\right) \\ &= \begin{cases} 2\left(\varepsilon_{\alpha_1\tau_i\gamma}\delta_{\eta\alpha_2} + \varepsilon_{\alpha_2\tau_j\eta}\delta_{\gamma\alpha_1}\right) & \text{if } \xi_i = \gamma,\ \xi_j = \eta, \\ 2\varepsilon_{\alpha_1\tau_i\gamma}\delta_{\tau_j\alpha_2} & \text{if } \xi_i = \gamma,\ \xi_j = \eta,\ \eta = \tau_j, \\ 0 & \text{else} \end{cases}, \end{aligned} \tag{9}$$

where $\eta, \gamma \in \{x, y, z\}$. Selecting other observable $O = \sigma^{(i)}_{\xi_i}$, we can rewrite (6)-(8) differently as

$$\begin{aligned} B^{(i)}_{\alpha_1}(\rho^{(i,j)}_{\tau_i,\tau_j}, \sigma^{(i)}_{\xi_i}) &= \varepsilon_{\alpha_1\tau_i\gamma}\delta_{\xi_i\gamma} = \begin{cases} \varepsilon_{\alpha_1\tau_i\gamma} & \text{if } \xi_i = \gamma, \\ 0 & \text{else} \end{cases}, \quad B^{(j)}_{\alpha_1}(\rho^{(i,j)}_{\tau_i,\tau_j}, \sigma^{(i)}_{\xi_i}) = 0, \\ B^{(ij)}_{\alpha_1\alpha_2}(\rho^{(i,j)}_{\tau_i,\tau_j}, \sigma^{(i)}_{\xi_i}) &= 2\varepsilon_{\alpha_1\tau_i\gamma}\delta_{\alpha_2\tau_j}\delta_{\xi_i\gamma} = \begin{cases} 2\varepsilon_{\alpha_1\tau_i\gamma}\delta_{\alpha_2\tau_j} & \text{if } \xi_i = \gamma, \\ 0 & \text{else} \end{cases}. \end{aligned} \tag{10}$$

Next, selecting $O = \sigma^{(j)}_{\xi_j}$, we can rewrite (6)-(8) as

$$\begin{aligned} B^{(i)}_{\alpha_1}(\rho^{(i,j)}_{\tau_i,\tau_j}, O) &= 0, \quad B^{(j)}_{\alpha_1}(\rho^{(i,j)}_{\tau_i,\tau_j}, \sigma^{(i)}_{\xi_i}) = \varepsilon_{\alpha_1\tau_j\zeta}\delta_{\xi_j\zeta} = \begin{cases} \varepsilon_{\alpha_1\tau_j\zeta} & \text{if } \xi_j = \zeta, \\ 0 & \text{else} \end{cases}, \\ B^{(ij)}_{\alpha_1\alpha_2}(\rho^{(i,j)}_{\tau_i,\tau_j}, \sigma^{(j)}_{\xi_j}) &= 2\varepsilon_{\alpha_2\tau_j\eta}\delta_{\alpha_1\tau_i}\delta_{\xi_j\eta} = \begin{cases} 2\varepsilon_{\alpha_2\tau_j\eta}\delta_{\alpha_1\tau_i} & \text{if } \xi_j = \eta, \\ 0 & \text{else} \end{cases}. \end{aligned} \tag{11}$$

For $k \geq 2$ we can write the general matrix element:

$$\begin{aligned} B^{(m_1,\ldots,m_k)}_{\alpha_1,\ldots,\alpha_k}(\rho^{(i,j)}_{\tau_i,\tau_j}, O) &= \frac{1}{2}\Bigg(\varepsilon_{\alpha_i\tau_i\gamma}\Big(\mathrm{tr}\Big[(\sigma^{(1)}_{\alpha_1} \otimes \cdots \otimes \sigma^{(i)}_\gamma \otimes \cdots \otimes \sigma^{(j)}_{\alpha_j} \otimes \cdots \otimes \sigma^{(k)}_{\alpha_k})O\Big] \\ &+ \delta_{\alpha_j\tau_j}\,\mathrm{tr}\Big[(\sigma^{(1)}_{\alpha_1} \otimes \cdots \otimes \sigma^{(i)}_\gamma \otimes \cdots \otimes I^{(j)} \otimes \cdots \otimes \sigma^{(k)}_{\alpha_k})O\Big]\Big) \\ &+ \varepsilon_{\alpha_j\tau_j\eta}\Big(\mathrm{tr}\Big[(\sigma^{(1)}_{\alpha_1} \otimes \cdots \otimes \sigma^{(i)}_{\alpha_i} \otimes \cdots \otimes \sigma^{(j)}_\eta \otimes \cdots \otimes \sigma^{(k)}_{\alpha_k})O\Big] \\ &+ \delta_{\alpha_i\tau_i}\,\mathrm{tr}\Big[(\sigma^{(1)}_{\alpha_1} \otimes \cdots \otimes I^{(i)} \otimes \cdots \otimes \sigma^{(j)}_\eta \otimes \cdots \otimes \sigma^{(k)}_{\alpha_k})O\Big]\Big)\Bigg), \end{aligned} \tag{12}$$

where O is acting on $(1,\dots,k)$ qubits. Let $O=\sigma^{(1)}_{\xi_1}\otimes\dots\otimes\sigma^{(k)}_{\xi_k}$, holds. Then we can rewrite (12) as follows

$$
\begin{aligned}
&B^{(1,\dots,k)}_{\alpha_1,\dots,\alpha_k}(\rho^{(i,j)}_{\tau_i,\tau_j},\sigma^{(1)}_{\xi_1}\otimes\dots\otimes\sigma^{(k)}_{\xi_k})\equiv -iTr\left([\sigma^{(1)}_{\alpha_1}\otimes\dots\otimes\sigma^{(k)}_{\alpha_k},\rho^{(i,j)}_{\tau_i,\tau_j}](\sigma^{(1)}_{\xi_1}\otimes\dots\otimes\sigma^{(k)}_{\xi_k})\right)\\
=\;& 2^{k-1}(\varepsilon_{\alpha_i\tau_i\gamma}\delta_{\alpha_1\xi_1}\dots\delta_{\gamma\xi_i}\dots\delta_{\alpha_j\xi_j}\dots\delta_{\alpha_k\xi_k}+\varepsilon_{\alpha_j\tau_j\eta}\delta_{\alpha_1\xi_1}\dots\delta_{\alpha_i\xi_i}\dots\delta_{\eta\xi_j}\dots\delta_{\alpha_k\xi_k}) \qquad (13)\\
=\;& \begin{cases} 2^{k-1}(\varepsilon_{\alpha_i\tau_i\gamma}\delta_{\eta\alpha_j}+\varepsilon_{\alpha_j\tau_j\eta}\delta_{\gamma\alpha_i}) & \text{if } \xi_i=\gamma,\ \xi_j=\eta,\ \xi_{1,\dots,k}=\alpha_{1,\dots,k},\\ 0 & \text{else}\end{cases}.
\end{aligned}
$$

From this result, for $k=3$ we get

$$B^{(i,j,l)}_{\alpha_i\alpha_j\alpha_l}(\rho^{(i,j)}_{\tau_i,\tau_j},\sigma^{(i)}_{\xi_i}\otimes\sigma^{(j)}_{\xi_j}\otimes\sigma^{(l)}_{\xi_l})=2(\varepsilon_{\alpha_i\tau_i\gamma}\delta_{\gamma,\xi_i}\delta_{\alpha_j\xi_j}+\varepsilon_{\alpha_j\tau_j\eta}\delta_{\alpha_i\xi_i}\delta_{\eta\xi_j})\delta_{\alpha_l\xi_l}. \qquad (14)$$

To isolate $\{L^{(m)}_{\mu\nu}\}$ we recall the assumption that ρ_{n-2} is the density matrix of the maximally mixed state. Then the Lindblad part of the equation (3) for an observable $O=\sigma^{(i)}_{\xi_i}\otimes\sigma^{(j)}_{\xi_j}$ is

$$
\begin{aligned}
\mathcal{L}^k_{\mu\nu}(\rho^{(i,j)}_{\tau_i,\tau_j},\sigma^{(i)}_{\xi_i}\otimes\sigma^{(j)}_{\xi_j}) &\equiv L^{(k)}_{\mu\nu}\,\mathrm{tr}\left[\left(\left(\sigma^{(k)}_\mu\rho^{(i,j)}_{\tau_i,\tau_j}\sigma^{(k)}_\nu-\frac{1}{2}\{\sigma^{(k)}_\nu\sigma^{(k)}_\mu,\rho^{(i,j)}_{\tau_i,\tau_j}\}\right)\right)(\sigma^{(i)}_{\xi_i}\otimes\sigma^{(j)}_{\xi_j})\right] \qquad (15)\\
&= \begin{cases} L^{(i)}_{\mu\nu}(2i\varepsilon_{\mu\nu\gamma}\delta_{\gamma\xi_i}+2\delta_{\nu\tau_i}\delta_{\mu\xi_i}-\frac{3}{2}\delta_{\mu\nu}\delta_{\xi_i\tau_i})\delta_{\tau_j\xi_j} & \text{if } k=i\\ L^{(j)}_{\mu\nu}(2i\varepsilon_{\mu\nu\gamma}\delta_{\gamma\xi_j}+2\delta_{\nu\tau_j}\delta_{\mu\xi_j}-\frac{3}{2}\delta_{\mu\nu}\delta_{\xi_j\tau_j})\delta_{\tau_i\xi_i} & \text{if } k=j\\ 0 & \text{else}\end{cases}.
\end{aligned}
$$

Let us substitute the conditions (9) in (15). We get the following results:

$$\mathcal{L}^i_{\mu\nu}(\rho^{(i,j)}_{\tau_i,\tau_j},\sigma^{(i)}_{\xi_i}\otimes\sigma^{(j)}_{\xi_j})=L^{(i)}_{\mu\nu}(2i\varepsilon_{\mu\nu\gamma}+2\delta_{\nu\tau_i}\delta_{\mu\gamma}-\frac{3}{2}\delta_{\mu\nu}\delta_{\gamma\tau_i})\delta_{\tau_j\eta}\quad \text{if } \xi_i=\gamma,\ \xi_j=\eta, \qquad (16)$$

and

$$\mathcal{L}^j_{\mu\nu}(\rho^{(i,j)}_{\tau_i,\tau_j},\sigma^{(i)}_{\xi_i}\otimes\sigma^{(j)}_{\xi_j})=L^{(j)}_{\mu\nu}(2i\varepsilon_{\mu\nu\gamma}\delta_{\gamma\eta}+2\delta_{\nu\tau_j}\delta_{\mu\eta}-\frac{3}{2}\delta_{\mu\nu}\delta_{\eta\tau_j})\delta_{\tau_i\gamma}\quad \text{if } \xi_i=\gamma,\ \xi_j=\eta. \qquad (17)$$

However, for an observable $O=\sigma^{(i)}_{\xi_i}$ the Lindblad part of the equation (3) is the following

$$
\begin{aligned}
\mathcal{L}^k_{\mu\nu}(\rho^{(i,j)}_{\tau_i,\tau_j},\sigma^{(i)}_{\xi_i}) &\equiv L^{(k)}_{\mu\nu}\,\mathrm{tr}\left[\left(\left(\sigma^{(k)}_\mu\rho^{(i,j)}_{\tau_i,\tau_j}\sigma^{(k)}_\nu-\frac{1}{2}\{\sigma^{(k)}_\nu\sigma^{(k)}_\mu,\rho^{(i,j)}_{\tau_i,\tau_j}\}\right)\right)(\sigma^{(i)}_{\xi_i})\right] \qquad (18)\\
&= \begin{cases} L^{(i)}_{\mu\nu}(2i\varepsilon_{\mu\nu\gamma}\delta_{\gamma\xi_i}+\delta_{\nu\tau_i}\delta_{\mu\xi_i}-\frac{3}{2}\delta_{\mu\nu}\delta_{\xi_i\tau_i}+\delta_{\mu\xi_i}\delta_{\tau_i\nu}) & \text{if } k=i\\ 0 & \text{if } k=j\\ 0 & \text{else}\end{cases}.
\end{aligned}
$$

Substituting the conditions (10) in (18), we get

$$\mathcal{L}^i_{\mu\nu}(\rho^{(i,j)}_{\tau_i,\tau_j},\sigma^{(i)}_{\xi_i}) \;=\; L^{(i)}_{\mu\nu}(2i\varepsilon_{\mu\nu\gamma}+2\delta_{\nu\tau_i}\delta_{\mu\gamma}-\frac{3}{2}\delta_{\mu\nu}\delta_{\gamma\tau_i}),\quad \text{if } \xi_i=\gamma. \qquad (19)$$

Next, for an observable $O=\sigma^{(i)}_{\xi_i}\otimes\sigma^{(j)}_{\xi_j}\otimes\sigma^{(l)}_{\xi_l}$ we can write

$$
\begin{aligned}
\mathcal{L}^k_{\mu\nu}(\rho^{(i,j)}_{\tau_i,\tau_j},\sigma^{(i)}_{\xi_i}\otimes\sigma^{(j)}_{\xi_j}\otimes\sigma^{(l)}_{\xi_l}) &\equiv L^{(k)}_{\mu\nu}\,\mathrm{tr}\left[\left(\left(\sigma^{(k)}_\mu\rho^{(i,j)}_{\tau_i,\tau_j}\sigma^{(k)}_\nu-\frac{1}{2}\{\sigma^{(k)}_\nu\sigma^{(k)}_\mu,\rho^{(i,j)}_{\tau_i,\tau_j}\}\right)\right)(\sigma^{(i)}_{\xi_i}\otimes\sigma^{(j)}_{\xi_j}\otimes\sigma^{(l)}_{\xi_l})\right]\\
&= \begin{cases} 2iL^{(l)}_{\mu\nu}\varepsilon_{\mu\nu\gamma}\delta_{\gamma\xi_l}\delta_{\tau_i\xi_i}\delta_{\tau_j\xi_j} & \text{if } k=l\\ 0 & \text{else.}\end{cases} \qquad (20)
\end{aligned}
$$

Then, according to the results of the previous subsection, we get

$$\mathcal{L}^l_{\mu\nu}(\rho^{(i,j)}_{\tau_i,\tau_j},\sigma^{(i)}_{\xi_i}\otimes\sigma^{(j)}_{\xi_j}\otimes\sigma^{(l)}_{\xi_l})=2iL^{(l)}_{\mu\nu}\varepsilon_{\mu\nu\gamma}\delta_{\gamma\alpha_l}\delta_{\tau_i\gamma}\delta_{\tau_j\eta}\quad \text{if } \xi_i=\gamma,\ \xi_j=\eta,\ \xi_l=\alpha_l. \qquad (21)$$

Finally, for an observable $O=\sigma^{(1)}_{\xi_1}\otimes\dots\sigma^{(i)}_{\xi_i}\dots\otimes\sigma^{(j)}_{\xi_j}\dots\otimes\sigma^{(k)}_{\xi_k}$, $k>3$, the Lindblad part of the equation (3) is the following

$$L^{(m)}_{\mu\nu}\,\mathrm{tr}\left[\left(\left(\sigma^{(m)}_\mu\rho^{(i,j)}_{\tau_i,\tau_j}\sigma^{(m)}_\nu-\frac{1}{2}\{\sigma^{(m)}_\nu\sigma^{(m)}_\mu,\rho^{(i,j)}_{\tau_i,\tau_j}\}\right)\right)(\sigma^{(1)}_{\xi_1}\otimes\dots\otimes\sigma^{(i)}_{\xi_i}\dots\otimes\sigma^{(j)}_{\xi_j}\dots\otimes\sigma^{(k)}_{\xi_k})\right]=0. \qquad (22)$$

A. Final Results

After we selected the different observable operators and defined the density matrix $\rho_0 = \rho^{(i)}_{\tau_i} \otimes \rho^{(j)}_{\tau_j} \otimes \frac{I^{2n-2}}{2^{2n-2}}$, where the i and j qubits are in the Pauli states, we are ready to isolate the desired coefficients. For an observable $O = \sigma^{(i)}_\gamma \otimes \sigma^{(j)}_\eta$ we can write the equation (3) as follows

$$\begin{aligned}\frac{d}{dt}\,\mathrm{tr}\left[\rho_t(\sigma^{(i)}_\gamma \otimes \sigma^{(j)}_\eta)\right]|_{t=0} &= -2\sum_{\alpha_1} a^{(i)}_{\alpha_1}\varepsilon_{\alpha_1\tau_i\gamma}\delta_{\eta\tau_j} - 2\sum_{\alpha_1} a^{(j)}_{\alpha_1}\varepsilon_{\alpha_1\tau_j\eta}\delta_{\gamma\tau_i} \\ &- 2\sum_{\alpha_1,\alpha_2} a^{(ij)}_{\alpha_1\alpha_2}\left(\varepsilon_{\alpha_1\tau_i\gamma}\delta_{\eta\alpha_2} + \varepsilon_{\alpha_2\tau_j\eta}\delta_{\gamma\alpha_1}\right) + \sum_{\mu,\nu}\Big(L^{(i)}_{\mu\nu}(2i\varepsilon_{\mu\nu\gamma} + 2\delta_{\nu\tau_i}\delta_{\mu\gamma} - \frac{3}{2}\delta_{\mu\nu}\delta_{\gamma\tau_i})\delta_{\tau_j\eta} \\ &+ L^{(j)}_{\mu\nu}(2i\varepsilon_{\mu\nu\gamma}\delta_{\gamma\eta} + 2\delta_{\nu\tau_j}\delta_{\mu\eta} - \frac{3}{2}\delta_{\mu\nu}\delta_{\eta\tau_j})\delta_{\tau_i\gamma}\Big).\end{aligned} \tag{23}$$

Selecting $\tau_j \neq \eta$, $\tau_i \neq \gamma$, we can isolate the coefficients of the type $a^{(ij)}_{\alpha_1\alpha_2}$ in (23), namely

$$\frac{d}{dt}\,\mathrm{tr}\left[\rho_t(\sigma^{(i)}_\gamma \otimes \sigma^{(j)}_\eta)\right]|_{t=0} = -2\sum_{\alpha_1,\alpha_2} a^{(ij)}_{\alpha_1\alpha_2}\left(\varepsilon_{\alpha_1\tau_i\gamma}\delta_{\eta\alpha_2} + \varepsilon_{\alpha_2\tau_j\eta}\delta_{\gamma\alpha_1}\right). \tag{24}$$

Let us call $\rho_{t\tau_i\tau_j}$ the density matrix evaluated by the Hamiltonian evolution from ρ_0. From (24) we can find $a^{(ij)}_{\alpha_1\alpha_2}$. To this end, we select $\gamma = y$, $\eta = y$ and four pairs $\tau_i, \tau_j \in \{z, x; z, z; x, x; x, z\}$ to get the system of equations

$$\begin{aligned}\frac{d}{dt}\,\mathrm{tr}\left[\rho_{tzx}(\sigma^{(i)}_y \otimes \sigma^{(j)}_y)\right]|_{t=0} &= 2(a^{(ij)}_{xy} - a^{(ij)}_{yz}),\ \frac{d}{dt}\,\mathrm{tr}\left[\rho_{tzz}(\sigma^{(i)}_y \otimes \sigma^{(j)}_y)\right]|_{t=0} = 2(a^{(ij)}_{xy} + a^{(ij)}_{yx}), \\ \frac{d}{dt}\,\mathrm{tr}\left[\rho_{txx}(\sigma^{(i)}_y \otimes \sigma^{(j)}_y)\right]|_{t=0} &= -2(a^{(ij)}_{yz} + a^{(ij)}_{zy}),\ \frac{d}{dt}\,\mathrm{tr}\left[\rho_{txz}(\sigma^{(i)}_y \otimes \sigma^{(j)}_y)\right]|_{t=0} = -2(a^{(ij)}_{yx} + a^{(ij)}_{zy}).\end{aligned} \tag{25}$$

Since $a^{(ij)}_{yz} = a^{(ij)}_{zy}$ and $a^{(ij)}_{xy} = a^{(ij)}_{yx}$, we can write

$$a^{(ij)}_{xy} = \frac{1}{4}\frac{d}{dt}\,\mathrm{tr}\left[\rho_{tzz}(\sigma^{(i)}_y \otimes \sigma^{(j)}_y)\right]|_{t=0}, \quad a^{(ij)}_{yz} = -\frac{1}{4}\frac{d}{dt}\,\mathrm{tr}\left[\rho_{txx}(\sigma^{(i)}_y \otimes \sigma^{(j)}_y)\right]|_{t=0}. \tag{26}$$

Selecting $\gamma = x$, $\eta = y$ and four pairs $\tau_i, \tau_j \in \{y, z; y, x; z, z; z, x\}$ to get the system of equations

$$\begin{aligned}\frac{d}{dt}\,\mathrm{tr}\left[\rho_{tyz}(\sigma^{(i)}_x \otimes \sigma^{(j)}_y)\right]|_{t=0} &= 2(a^{(ij)}_{xx} + a^{(ij)}_{zy}),\ \frac{d}{dt}\,\mathrm{tr}\left[\rho_{tyx}(\sigma^{(i)}_x \otimes \sigma^{(j)}_y)\right]|_{t=0} = 2(a^{(ij)}_{zy} - a^{(ij)}_{xz}), \\ \frac{d}{dt}\,\mathrm{tr}\left[\rho_{tzz}(\sigma^{(i)}_x \otimes \sigma^{(j)}_y)\right]|_{t=0} &= 2(a^{(ij)}_{xx} - a^{(ij)}_{yy}),\ \frac{d}{dt}\,\mathrm{tr}\left[\rho_{tzx}(\sigma^{(i)}_x \otimes \sigma^{(j)}_y)\right]|_{t=0} = -2(a^{(ij)}_{yy} + a^{(ij)}_{xz}).\end{aligned} \tag{27}$$

Hence

$$\begin{aligned}a^{(ij)}_{xx} &= \frac{1}{2}\frac{d}{dt}\,\mathrm{tr}\left[\rho_{tyz}(\sigma^{(i)}_x \otimes \sigma^{(j)}_y)\right]|_{t=0} + \frac{1}{4}\frac{d}{dt}\,\mathrm{tr}\left[\rho_{txx}(\sigma^{(i)}_y \otimes \sigma^{(j)}_y)\right]|_{t=0}, \\ a^{(ij)}_{xz} &= -\frac{1}{2}\frac{d}{dt}\,\mathrm{tr}\left[\rho_{tyx}(\sigma^{(i)}_x \otimes \sigma^{(j)}_y)\right]|_{t=0} - \frac{1}{4}\frac{d}{dt}\,\mathrm{tr}\left[\rho_{txx}(\sigma^{(i)}_y \otimes \sigma^{(j)}_y)\right]|_{t=0}, \\ a^{(ij)}_{yy} &= -\frac{1}{2}\frac{d}{dt}\,\mathrm{tr}\left[\rho_{tzz}(\sigma^{(i)}_x \otimes \sigma^{(j)}_y)\right]|_{t=0} + \frac{1}{2}\frac{d}{dt}\,\mathrm{tr}\left[\rho_{tyz}(\sigma^{(i)}_x \otimes \sigma^{(j)}_y)\right]|_{t=0} \\ &+ \frac{1}{4}\frac{d}{dt}\,\mathrm{tr}\left[\rho_{txx}(\sigma^{(i)}_y \otimes \sigma^{(j)}_y)\right]|_{t=0}.\end{aligned} \tag{28}$$

Selecting $\gamma = x$, $\eta = z$ and four pairs $\tau_i, \tau_j \in \{y, y; z, x; y, x; z, y\}$, we get

$$\begin{aligned}\frac{d}{dt}\,\mathrm{tr}\left[\rho_{tyy}(\sigma^{(i)}_x \otimes \sigma^{(j)}_z)\right]|_{t=0} &= -2(a^{(ij)}_{xx} - a^{(ij)}_{zz}),\ \frac{d}{dt}\,\mathrm{tr}\left[\rho_{tzx}(\sigma^{(i)}_x \otimes \sigma^{(j)}_z)\right]|_{t=0} = 2(a^{(ij)}_{xy} - a^{(ij)}_{yz}), \\ \frac{d}{dt}\,\mathrm{tr}\left[\rho_{tyx}(\sigma^{(i)}_x \otimes \sigma^{(j)}_z)\right]|_{t=0} &= -2(a^{(ij)}_{zz} - a^{(ij)}_{xy}),\ \frac{d}{dt}\,\mathrm{tr}\left[\rho_{tzy}(\sigma^{(i)}_x \otimes \sigma^{(j)}_z)\right]|_{t=0} = -2(a^{(ij)}_{xx} + a^{(ij)}_{yz}).\end{aligned} \tag{29}$$

Hence the last coefficient is

$$a^{(ij)}_{zz} = -\frac{1}{2}\frac{d}{dt}\,\mathrm{tr}\left[\rho_{tyx}(\sigma^{(i)}_x \otimes \sigma^{(j)}_z)\right]|_{t=0} + \frac{1}{4}\frac{d}{dt}\,\mathrm{tr}\left[\rho_{tzz}(\sigma^{(i)}_y \otimes \sigma^{(j)}_y)\right]|_{t=0}. \tag{30}$$

To find the other coefficients we select $\tau_j = \eta$, $\tau_i \neq \gamma$ and rerewrite (23) as

$$\frac{d}{dt}\,\mathrm{tr}\left[\rho_t(\sigma_\gamma^{(i)}\otimes\sigma_\eta^{(j)})\right]|_{t=0} = -2\sum_{\alpha_1} a_{\alpha_1}^{(i)}\varepsilon_{\alpha_1\tau_i\gamma} - 2\sum_{\alpha_1,\alpha_2} a_{\alpha_1\alpha_2}^{(ij)}\varepsilon_{\alpha_1\tau_i\gamma}\delta_{\eta\alpha_2} + 2\sum_{\mu=x,y,z} L_{\mu\mu}^{(i)}\delta_{\mu\tau_i}\delta_{\mu\gamma} + 2\sum_{\mu,\nu=x,y,z}^{\mu\neq\nu} L_{\mu\nu}^{(i)}(i\varepsilon_{\mu\nu\gamma} + \delta_{\nu\tau_i}\delta_{\mu\gamma}). \tag{31}$$

Next, for $\tau_j \neq \eta$, $\tau_i = \gamma$, we can rewrite (23) as

$$\frac{d}{dt}\,\mathrm{tr}\left[\rho_t(\sigma_\gamma^{(i)}\otimes\sigma_\eta^{(j)})\right]|_{t=0} = -2\sum_{\alpha_1} a_{\alpha_1}^{(j)}\varepsilon_{\alpha_1\tau_j\eta} - 2\sum_{\alpha_1,\alpha_2} a_{\alpha_1\alpha_2}^{(ij)}\varepsilon_{\alpha_2\tau_j\eta}\delta_{\gamma\alpha_1} + 2\sum_{\mu=x,y,z} L_{\mu\mu}^{(i)}\delta_{\mu\tau_j}\delta_{\mu\eta} + 2\sum_{\mu,\nu=x,y,z}^{\mu\neq\nu} L_{\mu\nu}^{(i)}(i\varepsilon_{\mu\nu\gamma}\delta_{\gamma\eta} + \delta_{\nu\tau_j}\delta_{\mu\eta}). \tag{32}$$

Selecting an observable $O = \sigma_\gamma^{(i)}$, we can write

$$\frac{d}{dt}\,\mathrm{tr}\left[\rho_t\sigma_\gamma^{(i)}\right]|_{t=0} = -\sum_{\alpha_1} a_{\alpha_1}^{(i)}\varepsilon_{\alpha_1\tau_i\gamma} - 2\sum_{\alpha_1,\alpha_2} a_{\alpha_1\alpha_2}^{(ij)}\varepsilon_{\alpha_1\tau_i\gamma}\delta_{\alpha_2\tau_j} + 2\sum_{\mu=x,y,z} L_{\mu\mu}^{(i)}(\delta_{\mu\tau_i}\delta_{\mu\gamma} - \frac{3}{4}\delta_{\gamma\tau_i}) + 2\sum_{\mu,\nu=x,y,z}^{\mu\neq\nu} L_{\mu\nu}^{(i)}(i\varepsilon_{\mu\nu\gamma} + \delta_{\nu\tau_i}\delta_{\mu\gamma}). \tag{33}$$

For the other observable $O = \sigma_\eta^{(j)}$, the result is the following

$$\frac{d}{dt}\,\mathrm{tr}\left[\rho_t\sigma_\eta^{(j)})\right]|_{t=0} = -\sum_{\alpha_1} a_{\alpha_1}^{(j)}\varepsilon_{\alpha_1\tau_j\eta} - 2\sum_{\alpha_1,\alpha_2} a_{\alpha_1\alpha_2}^{(ij)}\varepsilon_{\alpha_2\tau_j\eta}\delta_{\alpha_1\tau_i} + 2\sum_{\mu=x,y,z} L_{\mu\mu}^{(j)}(\delta_{\mu\tau_j}\delta_{\mu\eta} - \frac{3}{4}\delta_{\eta\tau_j}) + 2\sum_{\mu,\nu=x,y,z}^{\mu\neq\nu} L_{\mu\nu}^{(j)}(i\varepsilon_{\mu\nu\gamma}\delta_{\gamma\eta} + \delta_{\nu\tau_j}\delta_{\mu\eta}). \tag{34}$$

Substituting (33) in (31), we can write

$$\frac{d}{dt}\,\mathrm{tr}\left[\rho_t(\sigma_\gamma^{(i)}\otimes\sigma_\eta^{(j)})\right]|_{t=0} - \frac{d}{dt}\,\mathrm{tr}\left[\rho_t\sigma_\gamma^{(i)}\right]|_{t=0} = -\sum_{\alpha_1} a_{\alpha_1}^{(i)}\varepsilon_{\alpha_1\tau_i\gamma} + 2\sum_{\alpha_1,\alpha_2} a_{\alpha_1\alpha_2}^{(ij)}\varepsilon_{\alpha_1\tau_i\gamma}(\delta_{\tau_j\alpha_2} - \delta_{\eta\alpha_2}) + \frac{3}{2}\sum_{\mu=x,y,z} L_{\mu\mu}^{(i)}\delta_{\gamma\tau_i}. \tag{35}$$

Since in (31) the conditions $\tau_j = \eta$, $\tau_i \neq \gamma$, hold, we can rewrite the latter equation as

$$\frac{d}{dt}\,\mathrm{tr}\left[\rho_t(\sigma_\gamma^{(i)}\otimes\sigma_\eta^{(j)})\right]|_{t=0} - \frac{d}{dt}\,\mathrm{tr}\left[\rho_t\sigma_\gamma^{(i)}\right]|_{t=0} = -\sum_{\alpha_1} a_{\alpha_1}^{(i)}\varepsilon_{\alpha_1\tau_i\gamma}. \tag{36}$$

Solving the latter equation, we find $a_{\alpha_1}^{(i)}$. To this end, we select $\gamma = y$, $\tau_i = x$, $\tau_j = \eta \in \{x,y,z\}$ and get

$$a_x^{(i)} = -\frac{d}{dt}\,\mathrm{tr}\left[\rho_{tx\eta}(\sigma_y^{(i)}\otimes\sigma_\eta^{(j)})\right]|_{t=0} + \frac{d}{dt}\,\mathrm{tr}\left[\rho_{tx\eta}\sigma_y^{(i)}\right]|_{t=0}. \tag{37}$$

Next, for $\gamma = x$, $\tau_i = z$, $\tau_j = \eta \in \{x,y,z\}$ we get the solution of (36), namely

$$a_y^{(i)} = -\frac{d}{dt}\,\mathrm{tr}\left[\rho_{tz\eta}(\sigma_x^{(i)}\otimes\sigma_\eta^{(j)})\right]|_{t=0} + \frac{d}{dt}\,\mathrm{tr}\left[\rho_{tz\eta}\sigma_x^{(i)}\right]|_{t=0}. \tag{38}$$

Finally, for $\gamma = x$, $\tau_i = y$, $\tau_j = \eta \in \{x,y,z\}$ the solution is

$$a_z^{(i)} = \frac{d}{dt}\,\mathrm{tr}\left[\rho_{ty\eta}(\sigma_x^{(i)}\otimes\sigma_\eta^{(j)})\right]|_{t=0} - \frac{d}{dt}\,\mathrm{tr}\left[\rho_{ty\eta}\sigma_x^{(i)}\right]|_{t=0}. \tag{39}$$

Substituting (34) in (32), we can write

$$\frac{d}{dt}\,\mathrm{tr}\left[\rho_t(\sigma_\gamma^{(i)}\otimes\sigma_\eta^{(j)})\right]|_{t=0}-\frac{d}{dt}\,\mathrm{tr}\left[\rho_t\sigma_\eta^{(j)})\right]|_{t=0}=-\sum_{\alpha_1}a_{\alpha_1}^{(j)}\varepsilon_{\alpha_1\tau_j\eta}$$
$$+\ 2\sum_{\alpha_1,\alpha_2}a_{\alpha_1\alpha_2}^{(ij)}\varepsilon_{\alpha_2\tau_j\eta}(\delta_{\tau_i\alpha_1}-\delta_{\gamma\alpha_1})+\frac{3}{2}\sum_{\mu=x,y,z}L_{\mu\mu}^{(i)}\delta_{\eta\tau_j}. \qquad (40)$$

Since $\tau_j\neq\eta$, $\tau_i=\gamma$, hold, we can rewrite it as follows

$$\frac{d}{dt}\,\mathrm{tr}\left[\rho_t(\sigma_\gamma^{(i)}\otimes\sigma_\eta^{(j)})\right]|_{t=0}-\frac{d}{dt}\,\mathrm{tr}\left[\rho_t\sigma_\eta^{(j)})\right]|_{t=0}=-\sum_{\alpha_1}a_{\alpha_1}^{(j)}\varepsilon_{\alpha_1\tau_j\eta}. \qquad (41)$$

Solving the latter equation, we find $a_{\alpha_1}^{(j)}$. Selecting $\eta=y$, $\tau_j=z$ and $\tau_i=\gamma\in\{x,y,z\}$, we get

$$a_x^{(j)}=\frac{d}{dt}\,\mathrm{tr}\left[\rho_{t\gamma z}(\sigma_\gamma^{(i)}\otimes\sigma_y^{(j)})\right]|_{t=0}-\frac{d}{dt}\,\mathrm{tr}\left[\rho_{t\gamma z}\sigma_y^{(j)})\right]|_{t=0}. \qquad (42)$$

Selecting $\eta=x$, $\tau_j=z$ and $\tau_i=\gamma\in\{x,y,z\}$, we get

$$a_y^{(j)}=-\frac{d}{dt}\,\mathrm{tr}\left[\rho_{t\gamma z}(\sigma_\gamma^{(i)}\otimes\sigma_x^{(j)})\right]|_{t=0}+\frac{d}{dt}\,\mathrm{tr}\left[\rho_{t\gamma z}\sigma_x^{(j)})\right]|_{t=0}. \qquad (43)$$

Finally, selecting $\eta=y$, $\tau_j=x$ and $\tau_i=\gamma\in\{x,y,z\}$, we get the last coefficient of this type

$$a_z^{(j)}=-\frac{d}{dt}\,\mathrm{tr}\left[\rho_{t\gamma x}(\sigma_\gamma^{(i)}\otimes\sigma_y^{(j)})\right]|_{t=0}+\frac{d}{dt}\,\mathrm{tr}\left[\rho_{t\gamma x}\sigma_y^{(j)})\right]|_{t=0}. \qquad (44)$$

All the coefficients with the corresponding observables and initial states are given in Table I.

$a_\alpha^{(i)}$	$(O,\,\{\tau_i^{(i)},\tau_j^{(j)}\})$	Equation
$a_x^{(i)}$	$(\sigma_y^{(i)}\otimes\sigma_\eta^{(j)},\{x,\eta\})$; $(\sigma_y^{(i)},\{x,\eta\})$, $\eta\in\{x,y,z\}$	(37)
$a_y^{(i)}$	$(\sigma_x^{(i)}\otimes\sigma_\eta^{(j)},\{z,\eta\})$; $(\sigma_x^{(i)},\{z,\eta\})$, $\eta\in\{x,y,z\}$	(38)
$a_z^{(i)}$	$(\sigma_x^{(i)}\otimes\sigma_\eta^{(j)},\{y,\eta\})$; $(\sigma_x^{(i)},\{y,\eta\})$, $\eta\in\{x,y,z\}$	(39)
$a_x^{(j)}$	$(\sigma_\gamma^{(i)}\otimes\sigma_y^{(j)},\{\gamma,z\})$; $(\sigma_y^{(i)},\{\gamma,z\})$, $\gamma\in\{x,y,z\}$	(42)
$a_y^{(j)}$	$(\sigma_\gamma^{(i)}\otimes\sigma_x^{(j)},\{\gamma,z\})$; $(\sigma_x^{(i)},\{\gamma,z\})$, $\gamma\in\{x,y,z\}$	(43)
$a_z^{(j)}$	$(\sigma_\gamma^{(i)}\otimes\sigma_y^{(j)},\{\gamma,x\})$; $(\sigma_y^{(i)},\{\gamma,x\})$, $\gamma\in\{x,y,z\}$	(44)
$a_{xx}^{(ij)}$	$(\sigma_x^{(i)}\otimes\sigma_y^{(j)},\{y,z\})$; $(\sigma_y^{(i)}\otimes\sigma_y^{(j)},\{x,x\})$	(28)
$a_{yy}^{(ij)}$	$(\sigma_x^{(i)}\otimes\sigma_y^{(j)},\{z,z\})$; $(\sigma_x^{(i)}\otimes\sigma_y^{(j)},\{y,z\})$; $(\sigma_y^{(i)}\otimes\sigma_y^{(j)},\{x,x\})$	(28)
$a_{zz}^{(ij)}$	$(\sigma_x^{(i)}\otimes\sigma_z^{(j)},\{y,x\})$; $(\sigma_y^{(i)}\otimes\sigma_y^{(j)},\{z,z\})$	(30)
$a_{xy}^{(ij)}$	$(\sigma_y^{(i)}\otimes\sigma_y^{(j)},\{z,z\})$	(26)
$a_{yz}^{(ij)}$	$(\sigma_y^{(i)}\otimes\sigma_y^{(j)},\{x,x\})$	(26)
$a_{xz}^{(ij)}$	$(\sigma_x^{(i)}\otimes\sigma_y^{(j)},\{y,x\})$; $(\sigma_y^{(i)}\otimes\sigma_y^{(j)},\{x,x\})$	(28)

Supplementary Table I. The first column represents the type of the estimated Hamiltonian parameters $a_{\alpha_i}^{(i)}$, $a_{\alpha_i,\alpha_j}^{(ij)}$, $\alpha_i,\alpha_j\in\{x,y,z\}$. In the third column the number of equation for every parameter is provided, depending from the pairs of the observable O and the initial state $\rho_{\tau_i,\tau_j}^{(i,j)}=\rho_{\tau_i}^{(i)}\otimes\rho_{\tau_j}^{(j)}\otimes\rho_{n-2}$, $\tau_i,\tau_j=\{x,y,z\}$, given in the second column. For example, to estimate $a_x^{(i)}$ we use (37) with two pairs of observables and states: $\{O,\rho_{\tau_i,\tau_j}^{(i,j)}\}=\{(\sigma_y^{(i)}\otimes\sigma_\eta^{(j)},\rho_{x,\eta}^{(i,j)});(\sigma_y^{(i)},\rho_{x,\eta}^{(i,j)})\}$.

For an observable $O=\sigma_\gamma^{(i)}\otimes\sigma_\eta^{(j)}\otimes\sigma_{\alpha_l}^{(l)}$ we can write the equation

$$\frac{d}{dt}\,\mathrm{tr}\left[\rho_t(\sigma_\gamma^{(i)}\otimes\sigma_\eta^{(j)}\otimes\sigma_{\alpha_l}^{(l)})\right]|_{t=0}\ =\ -4\sum_{\alpha_i\alpha_j\alpha_l}a_{\alpha_i\alpha_j\alpha_l}^{(ijl)}(\varepsilon_{\alpha_i\tau_i\gamma}\delta_{\eta\alpha_j}+\varepsilon_{\alpha_j\tau_j\eta}\delta_{\gamma\alpha_i}) \qquad (45)$$
$$+\ 2i\sum_{\mu,\nu}L_{\mu\nu}^{(l)}\varepsilon_{\mu\nu\gamma}\delta_{\gamma\alpha_l}\delta_{\tau_i\gamma}\delta_{\tau_j\eta}.$$

Selecting $\tau_j=\eta$, $\tau_i\neq\gamma$, we can rewrite (45) as

$$\frac{d}{dt}\,\mathrm{tr}\left[\rho_t(\sigma_\gamma^{(i)}\otimes\sigma_\eta^{(j)}\otimes\sigma_{\alpha_l}^{(l)})\right]|_{t=0}=-4\sum_{\alpha_i\alpha_j\alpha_l}a_{\alpha_i\alpha_j\alpha_l}^{(ijl)}\varepsilon_{\alpha_i\tau_i\gamma}\delta_{\eta\alpha_j}. \qquad (46)$$

From this equation we can find $a^{(ijl)}_{\alpha_i\alpha_j\alpha_l}$. For an observable $O=\sigma^{(1)}_{\alpha_1}\otimes\ldots\sigma^{(i)}_{\gamma}\cdots\otimes\sigma^{(j)}_{\eta}\cdots\otimes\sigma^{(k)}_{\alpha_k}$, $k>3$ we can write

$$\frac{d}{dt}\,\mathrm{tr}\left[\rho_t(\sigma^{(1)}_{\alpha_1}\otimes\ldots\sigma^{(i)}_{\gamma}\cdots\otimes\sigma^{(j)}_{\eta}\cdots\otimes\sigma^{(k)}_{\alpha_k})\right]|_{t=0}=-2^{k-1}\sum_{\alpha_1,\ldots,\alpha_k}a^{(1\ldots k)}_{\alpha_1\ldots\alpha_k}(\varepsilon_{\alpha_i\tau_i\gamma}\delta_{\eta\alpha_j}+\varepsilon_{\alpha_j\tau_j\eta}\delta_{\gamma\alpha_i}). \tag{47}$$

Selecting $\tau_j=\eta$, $\tau_i\neq\gamma$, we can rewrite (47) as

$$\frac{d}{dt}\,\mathrm{tr}\left[\rho_t(\sigma^{(1)}_{\alpha_1}\otimes\ldots\sigma^{(i)}_{\gamma}\cdots\otimes\sigma^{(j)}_{\eta}\cdots\otimes\sigma^{(k)}_{\alpha_k})\right]|_{t=0}=-2^{k-1}\sum_{\alpha_1,\ldots,\alpha_k}a^{(1\ldots k)}_{\alpha_1\ldots\alpha_k}\varepsilon_{\alpha_i\tau_i\gamma}\delta_{\eta\alpha_j}. \tag{48}$$

Solving this equation, we find $a^{(1\ldots k)}_{\alpha_1\ldots\alpha_k}$.

From (15) we can find three Lindbladian coefficients. Selecting $\tau_i=y$, $\tau_j=\eta$, $\gamma=x$, we can find

$$L^{(i)}_{xy}=\frac{1}{2}\frac{d}{dt}\,\mathrm{tr}\left[\rho_{ty\eta}(\sigma^{(i)}_x\otimes\sigma^{(j)}_\eta)\right]|_{t=0}-a^{(i)}_z-a^{(ij)}_{z\eta}. \tag{49}$$

Next, for $\tau_i=z$, $\tau_j=\eta$, $\gamma=x$, we deduce

$$L^{(i)}_{xz}=\frac{1}{2}\frac{d}{dt}\,\mathrm{tr}\left[\rho_{tz\eta}(\sigma^{(i)}_x\otimes\sigma^{(j)}_\eta)\right]|_{t=0}+a^{(i)}_y+a^{(ij)}_{y\eta}. \tag{50}$$

Finally, for $\tau_i=z$, $\tau_j=\eta$, $\gamma=y$, the coefficient is

$$L^{(i)}_{yz}=\frac{1}{2}\frac{d}{dt}\,\mathrm{tr}\left[\rho_{tz\eta}(\sigma^{(i)}_y\otimes\sigma^{(j)}_\eta)\right]|_{t=0}-a^{(i)}_x-a^{(ij)}_{x\eta}. \tag{51}$$

From (23) we can find three more coefficients. Selecting $\tau_j=y$, $\tau_i=z$, $\gamma=z$, $\eta=x$, we get

$$L^{(j)}_{xy}=\frac{1}{2}\frac{d}{dt}\,\mathrm{tr}\left[\rho_{tzy}(\sigma^{(i)}_z\otimes\sigma^{(j)}_x)\right]|_{t=0}-a^{(j)}_z-a^{(ij)}_{zz}. \tag{52}$$

For $\tau_j=y$, $\tau_i=y$, $\gamma=y$, $\eta=z$, we can deduce

$$L^{(j)}_{zy}=\frac{1}{2}\frac{d}{dt}\,\mathrm{tr}\left[\rho_{tyy}(\sigma^{(i)}_y\otimes\sigma^{(j)}_z)\right]|_{t=0}+a^{(j)}_x+a^{(ij)}_{yx}. \tag{53}$$

Selecting $\tau_j=z$, $\tau_i=y$, $\gamma=y$, $\eta=x$, we get

$$L^{(j)}_{xz}=\frac{1}{2}\frac{d}{dt}\,\mathrm{tr}\left[\rho_{tyz}(\sigma^{(i)}_y\otimes\sigma^{(j)}_x)\right]|_{t=0}+a^{(j)}_y+a^{(ij)}_{yy}. \tag{54}$$

From (33) we find the following coefficients:

$$\begin{aligned}
L^{(i)}_{xx}&=\frac{1}{10}\left(-3\frac{d}{dt}\,\mathrm{tr}\left[\rho_{tx\tau_j}\sigma^{(i)}_x\right]|_{t=0}-3\frac{d}{dt}\,\mathrm{tr}\left[\rho_{ty\tau_j}\sigma^{(i)}_y\right]|_{t=0}+2\frac{d}{dt}\,\mathrm{tr}\left[\rho_{tz\tau_j}\sigma^{(i)}_z\right]|_{t=0}\right),\\
L^{(i)}_{yy}&=\frac{1}{10}\left(-3\frac{d}{dt}\,\mathrm{tr}\left[\rho_{tx\tau_j}\sigma^{(i)}_x\right]|_{t=0}+2\frac{d}{dt}\,\mathrm{tr}\left[\rho_{ty\tau_j}\sigma^{(i)}_y\right]|_{t=0}-3\frac{d}{dt}\,\mathrm{tr}\left[\rho_{tz\tau_j}\sigma^{(i)}_z\right]|_{t=0}\right),\\
L^{(i)}_{zz}&=\frac{1}{10}\left(2\frac{d}{dt}\,\mathrm{tr}\left[\rho_{tx\tau_j}\sigma^{(i)}_x\right]|_{t=0}-3\frac{d}{dt}\,\mathrm{tr}\left[\rho_{ty\tau_j}\sigma^{(i)}_y\right]|_{t=0}-3\frac{d}{dt}\,\mathrm{tr}\left[\rho_{tz\tau_j}\sigma^{(i)}_z\right]|_{t=0}\right).
\end{aligned} \tag{55}$$

From (34) the following coefficients can be found

$$\begin{aligned}
L^{(j)}_{xx}&=\frac{1}{10}\left(-3\frac{d}{dt}\,\mathrm{tr}\left[\rho_{t\gamma_1 x}\sigma^{(i)}_x\right]|_{t=0}-3\frac{d}{dt}\,\mathrm{tr}\left[\rho_{t\gamma_2 y}\sigma^{(i)}_y\right]|_{t=0}+2\frac{d}{dt}\,\mathrm{tr}\left[\rho_{t\gamma_3 z}\sigma^{(i)}_z\right]|_{t=0}\right),\\
L^{(j)}_{yy}&=\frac{1}{10}\left(-3\frac{d}{dt}\,\mathrm{tr}\left[\rho_{t\gamma_1 x}\sigma^{(i)}_x\right]|_{t=0}+2\frac{d}{dt}\,\mathrm{tr}\left[\rho_{t\gamma_2 y}\sigma^{(i)}_y\right]|_{t=0}-3\frac{d}{dt}\,\mathrm{tr}\left[\rho_{t\gamma_3 z}\sigma^{(i)}_z\right]|_{t=0}\right),\\
L^{(j)}_{zz}&=\frac{1}{10}\left(2\frac{d}{dt}\,\mathrm{tr}\left[\rho_{t\gamma_1 x}\sigma^{(i)}_x\right]|_{t=0}-3\frac{d}{dt}\,\mathrm{tr}\left[\rho_{t\gamma_2 y}\sigma^{(i)}_y\right]|_{t=0}-3\frac{d}{dt}\,\mathrm{tr}\left[\rho_{t\gamma_3 z}\sigma^{(i)}_z\right]|_{t=0}\right).
\end{aligned} \tag{56}$$

All the Lindbladian coefficients with the corresponding observables and initial states are given in Table II.

$L^{(i)}_{\mu\nu}$	$(O, \{\tau_i^{(i)}, \tau_j^{(j)}\})$	Equation
$L^{(i)}_{xx}$, $L^{(i)}_{yy}$, $L^{(i)}_{zz}$	$(\sigma_x^{(i)}, \{x, \tau_{j1}\})$; $(\sigma_y^{(i)}, \{y, \tau_{j2}\})$; $(\sigma_z^{(i)}, \{z, \tau_{j3}\})$, $\tau_{j1,2,3} \in \{x, y, z\}$	(55)
$L^{(j)}_{xx}$, $L^{(j)}_{yy}$, $L^{(j)}_{zz}$	$(\sigma_x^{(i)}, \{\gamma_1, x\})$; $(\sigma_y^{(i)}, \{\gamma_2, y\})$; $(\sigma_z^{(i)}, \{\gamma_3, z\})$, $\gamma_1 \in \{y, z\}$, $\gamma_2 \in \{x, z\}$, $\gamma_3 \in \{x, y\}$	(56)
$L^{(i)}_{xy}$	$(\sigma_x^{(i)} \otimes \sigma_\eta^{(j)}, \{y, \eta\})$, $\eta \in \{x, y, z\}$	(49)
$L^{(i)}_{xz}$	$(\sigma_x^{(i)} \otimes \sigma_\eta^{(j)}, \{z, \eta\})$, $\eta \in \{x, y, z\}$	(50)
$L^{(i)}_{yz}$	$(\sigma_y^{(i)} \otimes \sigma_\eta^{(j)}, \{z, \eta\})$, $\eta \in \{x, y, z\}$	(51)
$L^{(j)}_{xy}$	$(\sigma_z^{(i)} \otimes \sigma_x^{(j)}, \{z, y\})$	(52)
$L^{(j)}_{xz}$	$(\sigma_y^{(i)} \otimes \sigma_x^{(j)}, \{y, z\})$	(54)
$L^{(j)}_{yz}$	$(\sigma_y^{(i)} \otimes \sigma_z^{(j)}, \{y, y\})$	(53)

Supplementary Table II. The first column represents the type of the estimated Lindbladian parameters $L^{(i)}_{\mu\nu}$, $\mu, \nu \in \{x, y, z\}$. In the third column the number of equation for every parameter is provided, depending from the pairs of the observable O and the initial state $\rho^{(i,j)}_{\tau_i,\tau_j} = \rho^{(i)}_{\tau_i} \otimes \rho^{(j)}_{\tau_j} \otimes \rho_{n-2}$, $\tau_i, \tau_j = \{x, y, z\}$, given in the second column. For example, to estimate $L^{(j)}_{yz}$ we use (53) with one pair of observable and state: $\{O, \rho^{(i,j)}_{\tau_i,\tau_j}\} = \{\sigma_y^{(i)} \otimes \sigma_z^{(j)}, \rho^{(i,j)}_{y,y}\}$.

Supplementary Note 2: Numerical simulations

For simulations of the Hamiltonian learning protocol, we employed direct numerical solution of the time-dependent Schrodinger equation for the whole system of $n = 16$ qubits, whose time-dependent wavefunction $|\psi(t)\rangle$ is represented as an array of 2^n complex numbers, normalized to 1. The evolution includes both unitary component, governed by the system's Hamiltonian, and three non-unitary components: the first one, stemming from the quasi-static random frequency shifts, leading to essentially non-Markovian dephasing of the qubits with the characteristic time T_2^*, the second component, described as a set of Lindblad superoperators corresponding to the phase damping channel, leading to Markovian transverse decoherence of the qubits on the timescale T_2, and the third component, also leading to Markovian evolution of the qubit, and described as a set of Lindblad superoperators corresponding to the amplitude damping channel, which leads to longitudinal relaxation of the qubit on the timescale T_1. For precise meaning of the terms "Markovian" and "non-Markovian", see explanations below in subsection 2 B.

A. Unitary evolution

The simulation of the unitary (Hamiltonian) evolution was performed using the 2nd order Suzuki-Trotter decomposition of the evolution operator. The total Hamiltonian of the system in question can be written as

$$H = \sum_{m_1,m_2=1}^{n} \sum_{\alpha_1,\alpha_2=x,y} J_{m_1,m_2} \sigma_{\alpha_1}^{(m_1)} \sigma_{\alpha_2}^{(m_2)} + \frac{1}{2} \sum_{m_1=1}^{n} \Omega_{m_1} \sigma_z^{(m_1)}, \tag{57}$$

For the problem considered in this paper the couplings J_{m_1,m_2} are restricted to the nearest neighbor qubits on a 2−D lattice. Note that the actual frequency Ω_{m_1} of the m_1-th qubit in the Hamiltonian (57) is different from its nominal frequency $\tilde{\omega}_m$ mentioned in the main text; the reasons for this difference are explained in subsection 2 B below.

The Hamiltonian (57) is represented as a sum

$$H = H_X + H_Y + H_Z \tag{58}$$

$$H_X = \sum_{m_1,m_2=1}^{n} J_{m_1,m_2} \sigma_x^{(m_1)} \sigma_x^{(m_2)}, \quad H_Y = \sum_{m_1,m_2=1}^{n} J_{m_1,m_2} \sigma_y^{(m_1)} \sigma_y^{(m_2)}, \quad H_Z = \frac{1}{2} \sum_{m_1=1}^{n} \Omega_{m_1} \sigma_z^{(m_1)}, \tag{59}$$

and the corresponding Suzuki-Trotter decomposition of the evolution operator $U(\Delta t)$ for the (small) timestep of duration Δt has the form

$$U(\Delta t) \equiv \exp\left(-iH\Delta t\right) \approx \mathrm{e}^{-iH_Z\Delta t/2}\, \mathrm{e}^{-iH_Y\Delta t/2}\, \mathrm{e}^{-iH_X\Delta t}\, \mathrm{e}^{-iH_Y\Delta t/2}\, \mathrm{e}^{-iH_Z\Delta t/2}, \tag{60}$$

ensuring the overall time discretization error of the order $(\Delta t)^2$. The evolution operator over many time steps is a product of elementary operators $U(\Delta t)$.

Each term in the sum representing the Hamiltonian H_X (and, similarly, H_Y and H_Z) commutes with all other

terms, therefore

$$\exp\left(-iH_X\Delta t\right) = \prod_{m_1,m_2=1}^{n} \exp\left(-iJ_{m_1,m_2}\Delta t\, \sigma_x^{(m_1)}\sigma_x^{(m_2)}\right) = \prod_{m_1,m_2=1}^{n} \left[\cos\left(J_{m_1,m_2}\Delta t\right) - i\sigma_x^{(m_1)}\sigma_x^{(m_2)}\,\sin\left(J_{m_1,m_2}\Delta t\right)\right]. \tag{61}$$

Each term in this direct product acts on the wavefunction $|\psi(t)\rangle$ in a straightforward manner: the entries of the array that represents the wavefunction turn into linear combinations of themselves. Similar direct-product representation holds for H_Y and H_Z as well, such that the action of the total evolution operator $U(\Delta t)$ is easy to compute, without the need to calculate or store $2^N \times 2^N$ matrices.

In order to represent the situation where the non-initialized part of the system is in the completely mixed state, but avoid using the density matrix explicitly (which would imply dealing with $2^n \times 2^n$ matrix instead of the single array of the size 2^n), we represent the completely mixed state as a wavefunction with random entries [1, 2]. Specifically, we sampled the real and the imaginary parts of each entry of the corresponding wavefunction independently from Gaussian distribution with zero mean and unit variance, and then normalized the resulting wavefunction to one. In this way, for instance, the situation where the first and the second qubit are both initialized in the state $|0\rangle$, while the rest of the system is in completely mixed state, i.e. when the system's density matrix is

$$\rho = |0\rangle\langle 0| \otimes |0\rangle\langle 0| \otimes \frac{I_{n-2}}{2^{n-2}}, \tag{62}$$

where I_{n-2} is an identity matrix of the size $2^{n-2} \times 2^{n-2}$, is represented using the total wavefunction in the form

$$|\psi\rangle = |0\rangle \otimes |0\rangle \otimes |\psi_{n-2}^{(r)}\rangle, \tag{63}$$

where the random state $|\psi_{n-2}^{(r)}\rangle$ of the remaining $n-2$ qubits is generated as described above. Such an approximation provides high accuracy, of the order of $\exp(-n/2)$, due to the measure concentration phenomenon [3].

Further improvement in accuracy was achieved by averaging the values of the relevant observables over $M = 189$ independent realizations of the random wavefunction (as well as other random quantities, see below), which reduced the error by an additional factor of the order $\sim 1/\sqrt{M} \approx 0.07$. The accuracy was also independently controlled by estimating the variance in the calculated values of the observables, and ensuring that this variance remains much smaller than the statistical error caused by the shot noise produced by sampling the relevant observables for each qubit.

B. Non-unitary evolution

The first non-unitary component of the system's evolution, dephasing of the m_1-th qubit on the timescale T_{2,m_1}^*, caused by its random static frequency shift, is modeled by directly reproducing the underlying physical picture. Namely, we assumed that the actual frequency Ω_{m_1} of the m_1-th qubit, see Eq. 57, is a sum of two contributions: the nominal value $\tilde{\omega}_{m_1}$, and a random shift β_{m_1} that remains constant during the system's evolution. The values of β_{m_1} were independently sampled from Gaussian distributions with zero mean and variance $b_{m_1}^2$, which can be different for different qubits. The parameter b_{m_1} determines the dephasing time T_{2,m_1}^* of the m_1-th qubit: if this qubit were uncoupled from the rest of the system, then, after averaging over β_{m_1}, its transverse (x- and y-) components would undergo Gaussian decay with time dependence $\exp(-b_{m_1}^2 t^2/2)$, i.e. $b_{m_1} = \sqrt{2}/T_{2,m_1}^*$.

As mentioned above in subsection 2 A, the evolution of the system was repeated $M = 189$ times; each time we used different realizations of the set of the random frequency shifts β_{m_1} (as well as other random quantities, such as e.g. different realizations of the random wavefunction, also see below). Within this approach, for each particular realization of the parameters β_j, the evolution of the system is unitary, and can be simulated using the system's wavefunction as described in subsection 2 A above, while the non-unitary decay occurs due to averaging of the relevant observables over different realizations of the random parameters β_{m_1} (along with other random quantities).

Note that the dephasing caused by averaging over the static random frequency shifts, with its characteristic Gaussian-like decay of the density matrix elements, cannot be described via Lindblad operators. It is an example of non-Markovian evolution, in the sense that it cannot be described by a set of first-order differential equations (with respect to time t), which would include only current values of the (averaged) elements of the system's density matrix $\rho(t)$; in other words, the future values of the (averaged) density matrix elements, at times $t+s$ ($s > 0$), are not completely determined by their current values at the moment of time t. At the same time, the static noise processes $\beta_{m_1}(t)$ representing the random frequency shifts are, of course, Markovian random processes, sastisfying the Chapman-Kolmogorov equation.

The two other components of the non-unitary evolution, addressed below, are Markovian, and can be described using the Lindblad operators. However, in order to avoid dealing with the density matrix, these components were also modeled by employing the random processes and calculating the averages of the relevant observables.

The second non-unitary component of the evolution corresponds to the Markovian dephasing, and can be described via the set of Lindblad superoperators corresponding to the phase damping channel. For an isolated qubit, this would lead to exponential decay of the transverse components of the m_1-th qubit, having the form $\exp(-t/T_{2,j})$. This kind of dephasing, being Markovian, can be described by a set of first-order differential equations, generalizing the well-known Bloch-Redfield equations [4], which include only the current values of the elements of the system's density matrix $\rho(t)$, such that the future values of the density matrix elements, at times $t+s$ ($s>0$), are completely determined by their current values at the moment of time t.

This decay was modeled by taking the z-rotation of the m_1-th qubit produced by Ω_{m_1} (i.e., produced by the action of the operator $\exp(-iH_Z\Delta t)$ in Eq. 60), and adding to it another time-dependent rotation around the z-axis by the angle $\Lambda_{m_1}(t)$. For each time step of duration Δt, the values $\Lambda_{m_1}(t)$ were sampled randomly, indepedently of each other and of their previous values, from Gaussian distribution with zero mean and variance $4g_{m_1}^2\tau_{m_1}\Delta t$. This choice for the quantity $\Lambda(t)$ can be visualized as a rotation induced by a time-dependent frequency shift $\delta\omega_{m_1}(t)$, which is represented by an Ornstein-Uhlenbeck noise process with the correlation function $\langle\delta\omega_{m_1}(t)\,\delta\omega_{m_1}(t+s)\rangle = g_{m_1}^2\exp(-|s|/\tau_{m_1})$, in the limit where the correlation time τ_{m_1} is much smaller than Δt, while the magnitude g_{m_1} is large (formally, $\tau_{m_1}\to 0$ and $g_{m_1}\to\infty$), but the combination $g_{m_1}^2\tau_{m_1} = 1/T_{2,m_1}$ remains finite. For an isolated qubit, the average evolution under the influence of such Ornstein-Uhlenbeck noise $\delta\omega_{m_1}(t)$ is known [5] to produce exponential decay of the qubit's transverse (x- and y-) components with the decay time T_2. Again, for each particular realization of the time-dependent random process $\Lambda_{m_1}(t)$, the evolution of the system is unitary, and can be simulated using the system's wavefunction as described in subsection 2 A (provided, of course, that $\Delta t \ll T_{2,m_1}$, to ensure accuracy of the Suzuki-Trotter decomposition), while the non-unitary decay occurs due to averaging over different realizations of the noise.

The third non-unitary component, describing exponential relaxation of the m_1-th qubit towards the state $|0\rangle$ on a timescale T_{1,m_1}, was simulated in a similar manner, by representing the non-unitary evolution via averaging over many realizations of a random unitary evolution, employing the approach described in Ref. [6], with some modifications improving the accuracy. Namely, at each time step, we calculated the probability p_{m_1} for the m_1-th qubit to make a transition ("quantum jump") from the state $|1\rangle$ to the state $|0\rangle$; the corresponding value is $p_{m_1} = w_{m_1,1}\,\mu_{m_1}^2$, where $\mu_{m_1} = \sqrt{1-\exp(-\Delta t/T_{1,m_1})}$, and $w_{m_1,1}$ is the total probability of the system to be in the subspace corresponding to the j-th qubit in the state $|1\rangle$. This transition was implemented with the probability p_{m_1} at each time step: the part of the system's wavefunction corresponding to the m_1-th qubit in the state $|0\rangle$ was replaced by its complement, i.e. by the part corresponding to the m_1-th qubit in the state $|1\rangle$, multiplied by the factor μ_{m_1}, and the part of the wavefunction corresponding to the j-th qubit in the state $|1\rangle$ was set to zero. Alternatively, with the probability $1-p_{m_1}$ at each time step, the part of the wavefunction corresponding to the m_1-th qubit in the state $|1\rangle$ was multiplied by the factor $\exp[-(1/2)\,\Delta t/T_{1,m_1}]$, while its complement was left unchanged. These transformations were applied to the wavefunction in succession, for all qubits (for all $m_1 = 1,\dots n$), and the resulting modified wavefunction was normalized back to 1. Since all these transformations commute with the action of the operator $\exp(-iH_Z\Delta t)$ in Eq. 60, they were applied at the end of each unitary time-step evolution, after application of the operator $U(\Delta t)$ given by Eq. 60, in parallel with the action of the operators $\exp(-iH_Z\Delta t)$ or $\exp(-iH_Z\Delta t/2)$.

Note that this implementation corresponds to the application to the wavefunction of the Krauss operators E_0 or E_1 (see Ref. 7), describing the amplitude damping quantum channel, with the corresponding probabilities, where E_1 corresponds to the event of the "quantum jump", and E_0 corresponds to the absence of it.

Supplementary Note 3: Numerical simulation of superconducting qubit platform

From the discussion in the main text we are simulating a 2D grid of qubits that interact only with the nearest neighbours. The coupling between two neighbouring qubits through a coupler can be described by a Hamiltonian. In our notations it can be rewritten as

$$H = \sum_{k=i,j} a_z^{(k)} H_z^{(k)} + a_{xx}^{(i,j)} H_{xx}^{(i,j)} + a_{yy}^{(i,j)} H_{yy}^{(i,j)}, \tag{64}$$

where we introduce the notations

$$a_z^{(i)} = \frac{1}{2}\tilde{\omega}^{(i)}, \quad a_z^{(j)} = \frac{1}{2}\tilde{\omega}^{(j)}, \quad a_{xx}^{(i,j)} = a_{yy}^{(i,j)} = \frac{1}{2}\left[\frac{g_i g_j}{\Delta} + g_{ij}\right], \tag{65}$$
$$H_z^{(i)} = \sigma_z^{(i)}, \quad H_z^{(j)} = \sigma_z^{(j)}, \quad H_{xx}^{(i,j)} = \sigma_x^{(i)}\sigma_x^{(j)}, \quad H_{yy}^{(i,j)} = \sigma_y^{(i)}\sigma_y^{(j)}.$$

Thus, we define the 16 qubits $2D$ grid, where we generate ω_1, ω_2, ω_c, g_1, g_2, g_{12} from Gaussian distribution with mean and variance $\mathcal{N}(0,1)$. The parameters $a_z^{(i)}$, $a_z^{(j)}$, $a_{xx}^{(ij)}$, $a_{yy}^{(ij)}$ we estimate in our simulation and the rates of decay of T_1, T_2 and $T_2^{\star}$ are given in the Table III. The observables and initial states, isolating the desired coefficients $a_z^{(i)}$, $a_z^{(j)}$, $a_{xx}^{(i,j)}$

(ij)	$a_{xx}^{(ij)}, a_{yy}^{(ij)}$ [kHz]	(i)	$a_z^{(i)}$ [kHz]	T_1 [μs]	T_2 [μs]	$T_2^{\star}$ [μs]
1, 2	1.28112	1	1.73807	58.5227	65.9752	151.515
2, 3	-0.716875	2	-0.816877	60.0269	65.1704	166.667
3, 4	-0.956949	3	-1.0602	59.2424	64.6375	163.934
4, 5	-0.819328	4	-0.913223	61.0255	65.7397	149.254
1, 6	-1.1682	5	-1.23118	59.0545	66.0886	147.059
2, 7	-0.213057	6	-0.654699	60.0915	66.1118	151.515
3, 8	-0.563789	7	-0.514756	59.8856	65.1432	153.846
4, 9	1.74022	8	2.0817	61.0389	64.8252	158.73
5, 10	1.68348	9	-0.568581	60.5375	66.2155	158.73
6, 7	-1.51535	10	-0.710498	61.5036	65.389	149.254
7, 8	-0.729672	11	1.86153	59.8949	65.825	147.059
8, 9	1.6622	12	-2.03725	60.3777	65.1203	153.846
9, 10	-0.314438	13	-1.31695	57.5781	65.6052	156.25
11, 12	-0.475787	14	-0.902159	59.1881	65.8892	158.73
6, 11	1.3663	15	-0.202118	60.0283	66.2967	144.928
7, 12	-2.03531	16	0.136975	58.9397	66.0541	149.254
12, 13	-1.22632					
13, 8	-0.717182					
13, 14	-0.546421					
14, 9	1.90836					
14, 15	-0.781306					
11, 16	-0.358714					

Supplementary Table III. The first column represents the numbers of qubits whose coefficients $a_{\alpha_i,\alpha_j}^{(ij)}$, $\alpha_i, \alpha_j \in \{x,y,z\}$ are not zero, given in the second column. The fourth column contains $a_{\alpha_i}^{(i)}$, $\alpha_i \in \{x,y,z\}$, $i = 1,\ldots,16$. The rates of decay of T_1, T_2 and $T_2^{\star}$, corresponding to i's qubit are given in the fifth and six's columns, respectively.

and $a_{yy}^{(i,j)}$, are given in Table IV. One can see, that we need three starting states, namely $\rho_{01} = \rho_x^{(i)} \otimes \rho_x^{(j)} \otimes \frac{I^{2n-2}}{2^{2n-2}}$, $\rho_{02} = \rho_y^{(i)} \otimes \rho_z^{(j)} \otimes \frac{I^{2n-2}}{2^{2n-2}}$ and $\rho_{03} = \rho_z^{(i)} \otimes \rho_z^{(j)} \otimes \frac{I^{2n-2}}{2^{2n-2}}$ to isolate all four unknown coefficients. We measure the expectation values of observables in different times. Next, the time traces of these expectation values are fitted, using the polynomial interpolation method, and the derivatives estimation is preceded. Finally, using (39), (44), (28) and (28), the estimates of the coefficients $a_z^{(i)}$, $a_z^{(j)}$, $a_{xx}^{(i,j)}$, $a_{yy}^{(i,j)}$ for the pair of (i,j) are obtained. We repeat this process for all pairs of interacting qubits to obtain all coefficients of the Hamiltonian of the $2D$ grid.

In the presence of the noise, the observables and initial states required to isolate the Lindbladian coefficients are given in Table V. One can see, that we need three extra starting states in the presence of the Lindbladian noise, namely $\rho_{04} = \rho_z^{(i)} \otimes \rho_y^{(j)} \otimes \frac{I^{2n-2}}{2^{2n-2}}$, $\rho_{05} = \rho_y^{(i)} \otimes \rho_y^{(j)} \otimes \frac{I^{2n-2}}{2^{2n-2}}$ and $\rho_{06} = \rho_z^{(i)} \otimes \rho_x^{(j)} \otimes \frac{I^{2n-2}}{2^{2n-2}}$ to find $L_{\mu\nu}^{(i)}$, $\mu,\nu \in \{x,y,z\}$.

$a_{\alpha_i}^{(i)}$	$\{O,\, \rho_{\tau_i,\tau_j}^{(i,j)}\}$	Equation
$a_z^{(i)}$	$\{\sigma_x^{(i)} \otimes \sigma_z^{(j)}, \rho_{y,z}^{(i,j)}\}$; $\{\sigma_x^{(i)}, \rho_{y,z}^{(i,j)}\}$	(39)
$a_z^{(j)}$	$\{\sigma_x^{(i)} \otimes \sigma_y^{(j)}, \rho_{x,x}^{(i,j)}\}$; $\{\sigma_y^{(i)}, \rho_{x,x}^{(i,j)}\}$	(44)
$a_{xx}^{(ij)}$	$\{\sigma_x^{(i)} \otimes \sigma_y^{(j)}, \rho_{y,z}^{(i,j)}\}$; $\{\sigma_y^{(i)} \otimes \sigma_y^{(j)}, \rho_{x,x}^{(i,j)}\}$	(28)
$a_{yy}^{(ij)}$	$\{\sigma_x^{(i)} \otimes \sigma_y^{(j)}, \rho_{z,z}^{(i,j)}\}$; $\{\sigma_x^{(i)} \otimes \sigma_y^{(j)}, \rho_{y,z}^{(i,j)}\}$; $\{\sigma_y^{(i)} \otimes \sigma_y^{(j)}, \rho_{x,x}^{(i,j)}\}$	(28)

Supplementary Table IV. In this table the minimal selection of the pairs $\{O,\, \rho_{\tau_i,\tau_j}^{(i,j)}\}$ is presented for our specific example. The first column represents the type of the estimated Hamiltonian (64) parameters $a_{\alpha_i}^{(i)}$, $a_{\alpha_i,\alpha_j}^{(ij)}$, $\alpha_i, \alpha_j \in \{x,y,z\}$. In the third column the number of equation for every parameter is provided, depending from the pairs of the observable O and the initial state $\rho_{\tau_i,\tau_j}^{(i,j)} = \rho_{\tau_i}^{(i)} \otimes \rho_{\tau_j}^{(j)} \otimes \rho_{N-2}$, $\tau_i, \tau_j = \{x,y,z\}$, given in the second column. To estimate all four parameters we need only three initial states: $\rho_{x,x}^{(i,j)}$, $\rho_{z,z}^{(i,j)}$ and $\rho_{y,z}^{(i,j)}$.

$L^{(i)}_{\mu\nu}$	$\{O,\, \rho^{(i,j)}_{\tau_i,\tau_j}\}$	Equation
$L^{(i)}_{xx}$, $L^{(i)}_{yy}$, $L^{(i)}_{zz}$	$\{\sigma^{(i)}_x, \rho^{(i,j)}_{x,x}\}$; $\{\sigma^{(i)}_y, \rho^{(i,j)}_{y,z}\}$; $\{\sigma^{(i)}_z, \rho^{(i,j)}_{z,z}\}$	(55)
$L^{(j)}_{xx}$, $L^{(j)}_{yy}$, $L^{(j)}_{zz}$	$\{\sigma^{(i)}_x, \rho^{(i,j)}_{z,x}\}$; $\{\sigma^{(i)}_y, \rho^{(i,j)}_{z,y}\}$; $\{\sigma^{(i)}_z, \rho^{(i,j)}_{y,z}\}$	(56)
$L^{(i)}_{xy}$	$\{\sigma^{(i)}_x \otimes \sigma^{(j)}_z, \rho^{(i,j)}_{y,z}\}$	(49)
$L^{(i)}_{xz}$	$\{\sigma^{(i)}_x \otimes \sigma^{(j)}_\eta, \rho^{(i,j)}_{z,z}\}$	(50)
$L^{(i)}_{yz}$	$\{\sigma^{(i)}_y \otimes \sigma^{(j)}_\eta, \rho^{(i,j)}_{z,z}\}$	(51)
$L^{(j)}_{xy}$	$\{\sigma^{(i)}_z \otimes \sigma^{(j)}_x, \rho^{(i,j)}_{z,y}\}$	(52)
$L^{(j)}_{xz}$	$\{\sigma^{(i)}_y \otimes \sigma^{(j)}_x, \rho^{(i,j)}_{y,z}\}$	(54)
$L^{(j)}_{yz}$	$\{\sigma^{(i)}_y \otimes \sigma^{(j)}_z, \rho^{(i,j)}_{y,y}\}$	(53)

Supplementary Table V. In this table the minimal selection of the pairs $\{O,\, \rho^{(i,j)}_{\tau_i,\tau_j}\}$ is presented for our specific example. The first column represents the type of the estimated Lindbladian parameters $L^{(i)}_{\mu\nu}$, $\mu,\nu \in \{x,y,z\}$. In the third column the number of equation for every parameter is provided, depending from the pairs of the observable O and the initial state $\rho^{(i,j)}_{\tau_i,\tau_j} = \rho^{(i)}_{\tau_i} \otimes \rho^{(j)}_{\tau_j} \otimes \rho_{N-2}$, $\tau_i, \tau_j = \{x,y,z\}$, given in the second column. To estimate all parameters we need only three extra states to the ones given by the previous table, namely: $\rho^{(i,j)}_{z,x}$, $\rho^{(i,j)}_{z,y}$ and $\rho^{(i,j)}_{y,y}$.

Supplementary Note 4: Approximating local time evolutions by polynomials

One of the main points behind our method is the fact that the time evolution of local observables at constant times is well-approximated by polynomials. The purpose of this section is to make this assertion precise.

Before we do that, let us set some notation. Given a system of n qubits on a D dimensional lattice Γ, we let $\mathcal{L}_\Lambda : \mathcal{M}_{2^n} \to \mathcal{M}_{2^n}$ be a Lindbladian which models the time evolution of the system in the Heisenberg picture. Note that in the supplementary information we consider a slightly more general class of evolutions than in the main text. There, we restricted to evolutions whose Hamiltonians were short range with two-body interactions and the noise acted on at most one qubit at a time. Here, in contrast, we will also consider k-local evolutions with long range.

We will assume that this Lindbladian can be written as:

$$\mathcal{L}_\Lambda = \sum_{A \subset \Lambda} \mathcal{L}_A, \tag{66}$$

where $\mathcal{L}_A$ is a Lindbladian only acting on the qubits in A. Given some graph $G = (V, E)$ on n vertices, we will say that $\mathcal{L}$ is $k-$local if $\mathcal{L}_A \neq 0$ only if A is a subset of vertices of G containing at most k vertices. Furthermore, we will say that $\mathcal{L}_\Lambda$ is locally bounded if there is a constant $g > 0$ such that for all $B \subset \Lambda$ we have that:

$$\| \sum_{A \subset \Lambda : A \cap B \neq \varnothing} \mathcal{L}_A \| \leq g|B|. \tag{67}$$

This condition is satisfied if e.g. $\mathcal{L}$ is a local Lindbladian on a $D-$dimensional lattice. In that case, we have $g = \mathcal{O}(D)$. However, this condition is also fulfilled for generators with algebraically decaying tails that decay at least like $x^{-(D+\delta)}$ for some $\delta > 0$. Moreover, for ease of notation we will let for a region $B \subset \Lambda$

$$\mathcal{L}_B = \sum_{A \subset B} \mathcal{L}_A \tag{68}$$

be the generator restricted or truncated to a subregion B.

Furthermore, given the lattice Λ, some region $X \subset V$ and $r > 0$, we will denote by $\Lambda_r(X)$ the set of vertices that are a distance at most r from X:

$$\Lambda_r(X) = \{v \in V : \exists x \in X \text{ s.t. } \operatorname{dist}(x, v) \leq r\}. \tag{69}$$

Here the distance is according to the lattice.

We will also require some norms for superoperators. Given a superoperator $\Phi : \mathcal{M}_{2^n} \to \mathcal{M}_{2^n}$ we define for $p, q \geq 1$

$$\|\Phi\|_{p \to q} = \sup_{X \in \mathcal{M}_{2^n}} \frac{\|\Phi(X)\|_q}{\|X\|_p}, \tag{70}$$

where $\|\cdot\|_p$ corresponds to the Schatten p-norm. Also note that $p = \infty$ corresponds to the operator norm, for which we will often drop the $+\infty$ and only write as $\|\cdot\|$. We will also consider completely bounded versions of these norms,

which are given by:

$$\|\Phi\|_{p\to q,cb} = \sup_{d\geq 1}\sup_{X\in\mathcal{M}_{2^n}\otimes\mathcal{M}_d}\frac{\|\Phi\otimes\mathrm{id}_d(X)\|_q}{\|X\|_p}. \tag{71}$$

It is then simple to see that derivatives of locally bounded evolutions can only increases with the size of the region they are defined on:

Lemma 4.1 (Derivatives of local evolutions)**.** *Let $\mathcal{L}_\Lambda$ be a locally bounded Lindbladian with constant g. For an observable O such that $\|O\|\leq 1$, an initial state ρ and a region $B\subset\Lambda$ defines $f_B:\mathbb{R}^+\to\mathbb{R}$ as $f_B(t)=\mathrm{tr}\left[e^{t\mathcal{L}_B}(O)\rho\right]$. Then for all $t\geq 0$:*

$$\left|f_B^{(k)}(t)\right| \leq (g|B|)^k\,. \tag{72}$$

In particular, for any $0<t<t_{\max}$ we have that:

$$\left|f_B(t)-\sum_{k=0}^{d}\frac{f_B^{(k)}(0)}{k!}t^k\right| \leq \frac{(t_{\max}g|B|)^{d+1}}{(d+1)!}. \tag{73}$$

Proof. The proof is elementary. Note that:

$$f_B^{(k)}(t) = \mathrm{tr}\left[e^{\mathcal{L}_B}((t\mathcal{L}_B)^k(O))\rho\right]. \tag{74}$$

Now, by Hölder's inequality we have that:

$$\left|f_B^{(k)}(t)\right| \leq \|e^{t\mathcal{L}_B}((t\mathcal{L}_B)^k(O))\|_\infty\|\rho\|_1 \overset{(1)}{\leq} t^k\|e^{t\mathcal{L}_B}\|_{\infty\to\infty}\|\mathcal{L}_B\|_{\infty\to\infty}^k \overset{(2)}{\leq} g^k|B|^k,$$

where in (1) we used the submultiplicativity of the operator norm, i.e. $\|\Phi_1\Phi_2\|_{\infty\to\infty}\leq\|\Phi_1\|_{\infty\to\infty}\|\Phi_2\|_{\infty\to\infty}$ for all linear maps Φ_1,Φ_2. In (2) we used the fact that for any quantum channel $\|e^{t\mathcal{L}}\|_{\infty\to\infty}=1$ and the fact that the Lindbladian is locally bounded with constant g. The estimate in Eq. (73) then immediately follows from Taylor's remainder theorem. □

We then immediately have:

Corollary 4.1. *In the same setting as Lemma 4.1 it holds that for any given $\epsilon>0$ and $t_{\max}>0$ s.t.*

$$\log\log(\epsilon^{-1})2et_{\max}g|B|\geq 1 \tag{75}$$

there is a polynomial p of degree

$$d = 2et_{\max}g|B|\log(\epsilon^{-1})-1 \tag{76}$$

such that for all $0\leq t\leq t_{\max}$ we have that

$$|f_B(t)-p(t)|\leq\epsilon. \tag{77}$$

Proof. It follows from Sitrling's approximation that the error in Eq. (73) is bounded by

$$\left|f(t)-\sum_{k=0}^{d}\frac{f^{(k)}(0)}{k!}t^k\right| \leq \frac{1}{\sqrt{2\pi d}}\left(\frac{et_{\max}g|B|}{d+1}\right)^{d+1}. \tag{78}$$

It is then easy to see that picking $d=2et_{\max}g|B|\log(\epsilon^{-1})-1$ is sufficient to ensure that the error in (73) is at most ϵ. Indeed, plugging in the value of d into Eq. (78), we get:

$$\left|f(t)-\sum_{k=0}^{d}\frac{f^{(k)}(0)}{k!}t^k\right| \leq \frac{1}{\sqrt{2\pi d}}\left(\frac{1}{\log(\epsilon^{-1})}\right)^{2et_{\max}g|B|\log(\epsilon^{-1})} = \tag{79}$$

$$\frac{1}{\sqrt{2\pi d}}\exp\left[-\log(\log(\epsilon^{-1}))\log(\epsilon^{-1})2et_{\max}g|B|\right] = \frac{1}{\sqrt{2\pi d}}\epsilon^{\log\log(\epsilon^{-1})2et_{\max}g|B|}\leq\epsilon. \tag{80}$$

Thus, the truncated Taylor expansion yields the desired polynomial. □

We conclude from Lemma 4.1 and Cor. 4.1 that local time evolutions are well-approximated by polynomials whose degree grows like the size of the region times the maximal time of evolution.

Also note that the estimate in Eq. (79) is quite loose and shows that for d as in Eq. (76) the error decays like a polynomial of high-degree in ϵ. But that rough approximation will be sufficient for our purposes.

Cor. 4.1 is an important step to prove our Thm. 4.1, but still does not correspond to the exact statement we wish to prove. This is because Cor. 4.1 is a statement about the *local* evolution, whereas Thm. 4.1 is a statement about the *global* evolution being well-approximated by a polynomial of small degree. The strategy to go from the local to the global evolution, is to show that for the (local) observables required for our protocl, the local evolution approximates the global one well.

Our main tool to show this approximatability of expectation values are Lieb-Robinson bounds [8–11], which exactly give conditions under which the local time evolution and the global one are close for small enough times and local observables. In order to provide a self-contained presentation, we include a brief introduction to Lieb-Robinson bounds in Sec. 7 of this appendix.

In fact, there are various ways of quantifying that this idea of local approximability and, thus, LR-bounds come in various forms. The version that we are going to work with here and which is discussed in detail in Sec. 7, considers an observable O_Y initially supported on in a region Y. For a region $B \supset Y$ we set $R = \mathrm{dist}(\Lambda\backslash\{B\}, Y)/k$, where k is the locality of the generator, then it is shown in Lemma 7.1 that indeed

$$\|(e^{t\mathcal{L}_\Lambda} - e^{t\mathcal{L}_{\Lambda_r(Y)}})(O_Y)\|_\infty \leq (e^{vt} - 1)h(R), \tag{81}$$

where $h : \mathbb{R}^+ \to \mathbb{R}^+$ is a monotonically increasing function such that $\lim_{R\to+\infty} h(R) = 0$ and v is some constant that depends on the generator, usually called the LR-velocity. On the other hand, the decay of the function h typically depends on how fast the interactions in the system decays spatially (i.e. if it is strictly local, exponentially decaying in the distance or even algebraically decaying) and the geometry of the underlying lattice. However, the important point for our purposes is that it does not depend on the system's size. For the specific case of short-range Hamiltonians discussed in the main text, we have that $h(R) = e^{-\mu R}$ for some constant $\mu > 0$. For algebraically decaying evolutions we usually have $h(x) = x^k$ for some $k \in \mathbb{R}^+$.

We refer again to Sec. 7 for a discussion of various LR-bounds available in the literature. But from Eq. (81) we immediately conclude that the values of the expectation values of global and local evolutions are well-approximated by each other. More precisely:

Proposition 4.1. *Let O_Y be an observable supported on some region Y, $\epsilon > 0$ and $t_{\max}$ be given. Assume Eq. (81) holds for the time evolution $\mathcal{L}_\Lambda$ and a function h. Let $l > 0$ be given by*

$$l = h^{-1}\left(\frac{\epsilon}{e^{vt_{\max}} - 1}\right). \tag{82}$$

Then we have for $\Lambda_l(Y)$ and all $0 < t < t_{\max}$ and any initial state ρ that

$$|\,\mathrm{tr}\left[\left(e^{t\mathcal{L}_\Lambda} - e^{t\mathcal{L}_{\Lambda_l(Y)}}\right)(O_Y)\rho\right]| \leq \epsilon. \tag{83}$$

Proof. The claim follows directly from Eq. (81) or Lemma 7.1 and a Hölder inequality. Indeed, for the value of l in Eq. (83), we obtain from Eq. (81) after some simplification that

$$\|(e^{t\mathcal{L}_\Lambda} - e^{t\mathcal{L}_{\Lambda_r(Y)}})(O_Y)\|_\infty \leq \epsilon. \tag{84}$$

□

Note that in the case of short-range systems we have that $h^{-1}(x) = \mu^{-1}\log(x)$. From now on we will suppress the terms of order $\log(\log(\epsilon^{-1}))$ or higher from the equations and denote bounds where we do this with $\tilde{\mathcal{O}}$. Thus, combining 4.1 with Prop. 4.1 we conclude that:

Theorem 4.1. *Let $\mathcal{L}_\Lambda$ be a locally bounded Lindbladian on a D-dimensional regular lattice with constant g. Moreover, let $t_{\max}, \epsilon > 0$ be given and O_Y is an observable such that $\|O_Y\| \leq 1$ and O_Y is supported on a constant number of qubits. Assume that $\mathcal{L}_\Lambda$ satisfies Eq. (81). Then there is a polynomial p of degree*

$$d = \tilde{\mathcal{O}}\left[\left(h^{-1}\left(\frac{\epsilon}{e^{vt_{\max}} - 1}\right)\right)^D t_{\max}\log(\epsilon^{-1})\right] \tag{85}$$

such that for all $0 \leq t \leq t_{\max}$:

$$\left|\mathrm{tr}\left[e^{t\mathcal{L}_\Lambda}(O_Y)\rho\right] - p(t)\right| \leq \epsilon, \tag{86}$$

and

$$p'(0) = \mathrm{tr}\left[\mathcal{L}_\Lambda(O_Y)\rho\right]. \tag{87}$$

Proof. It follows from Prop. 4.1 that a region of radius

$$l = \tilde{\mathcal{O}}\left[h^{-1}\left(\frac{\epsilon}{2(e^{t\max}-1)}\right)\right] \tag{88}$$

is enough to approximate the time evolution of $e^{t\mathcal{L}_\Lambda}(O_Y)$ up to $\epsilon/2$. If the original region Y has a constant number of qubits, then for a D-dimensional lattice we have $|\Lambda_l(Y)| = \mathcal{O}(l^D)$. It then follows from Cor. 4.1 that for regions of this size, it is sufficient to pick a degree that is

$$\mathcal{O}\left[h^{-1}\left(\frac{\epsilon}{2(e^{t\max}-1)}\right)^D t_{\max}\log(\epsilon^{-1}))\right] \tag{89}$$

to approximate the expectation value of the local evolution up to an error $\epsilon/2$. This concludes the proof by a triangle inequality. Eq. (87) is clear from properties of the truncated Taylor series. □

Thus, we see that as long as the time evolution of the system satisfies a Lieb-Robinson bound, we can approximate the expectation value of a local observable as a function of time by a polynomial whose degree is dictated by how fast the function h decays and the maximal time of the evolution. In particular, for $\epsilon^{-1} = \mathcal{O}(1)$ and time $t_{\max} = \mathcal{O}(1)$, we conclude that the degree of the polynomial is independent of the system's size.

In Thm. 4.1 we established that we can approximate the function $f : t \mapsto \mathrm{tr}\left[e^{t\mathcal{L}_\Lambda}(O_Y)\rho\right]$ well by a polynomial for constant times. However, to estimate the parameters of the Hamiltonian we are ultimately interested in the derivative of f at time 0. We will later show in Sec. 5 that for the special case of polynomials of bounded degree, a good recovery of the polynomial also implies a good recovery of the derivative. In particular, as for the polynomial in Lemma 4.1 we have that $p'(0) = f'(0)$, it is sufficient to argue that any two polynomials that approximates the curve $f(t)$ up to sufficiently large precision in a sufficiently large number of points must have close derivatives at 0 as well. This will be the subject of the next section and proved in Prop. 5.1.

Supplementary Note 5: (Robust) polynomial interpolation and derivative estimation

In this section of the appendix, we are going to review a result in the literature [12] that shows how to perform polynomial interpolation in a robust way even in the presence of outliers. Furthermore, we will show that good polynomial interpolation also implies a good approximation of derivatives of the polynomial, which is our end goal. We will use and review the results and algorithms of [12] for the robust polynomial interpolation and resort to Markov brothers' inequality [13] for estimating the error on the derivatives.

Let us start by briefly recalling the technical problems we wish to overcome. We assume we are able to approximate the expectation value of $f(t) = \mathrm{tr}\left[\rho e^{t\mathcal{L}}(O)\right]$ for some suitably-picked initial state ρ and time-evolved observable O. As argued in Sec. 4, the function f is well-approximated by a low degree polynomial p whenever the time evolution is generated by a local Lindbladian. Moreover, as shown in Sec. 1, by suitably choosing the observable and initial state, we can easily read off the value of the coupling of the Hamiltonian from the value of $f'(0)$. Thus, our goal is to find a polynomial p that approximates f from values of $f(t_i)$ for some $t_i \in [a, b]$ for then use p to infer $f'(0)$.

It is well-known that if p is a polynomial of degree d, then it is uniquely determined by its values at $d+1$ points. Thus, one could naively expect that having access to $f(t_1), \ldots, f(t_{\tilde{d}})$ points for $\tilde{d} \sim d$ times is sufficient to reconstruct f.

However, the present situation exhibits three challenges that need to be overcome to ensure that we can reliably apply polynomial interpolation methods and recover p from points $f(t_i)$:

1. we can only estimate $f(t)$, and not $p(t)$. And the value of f only approximates that of p up to some error ϵ_a, as discussed in Thm. 4.1.

2. we do not have access to the value of $f(t)$ directly, but can only approximate it to a precision ϵ_s by sampling from the output of the device at time t $\mathcal{O}(\epsilon_s^{-2})$ times.

3. we are interested in the value of $p'(0)$ and not in the polynomial p itself. Thus, we need to ensure a small error in estimating the derivative.

To deal with the first two problems the polynomial interpolation technique we use has to be robust to the noise stemming from both the approximation error from the polynomial approximation and the statistical noise. To deal with the third issue, we will show that we need to pick the final and initial time of the interpolation in a judicious manner.

To obtain some intuition about how to pick the times, let us consider the case of estimating the derivative of a quadratic polynomial at 0. I.e., if we have two linear functions $p, \hat{p}$ that are ϵ close in some interval $[a, b]$, how well can we infer the derivative of p at 0 from that of $\hat{p}$? First, note that if the interval $[a, b]$ is very small, then two functions can differ by ϵ and their derivatives can still differ by $\epsilon/(b-a)$ even for linear functions. This indicates we probably do not want to pick $b-a$ too small. However, as we know that increasing b also implies that, in our setting, we need to increase the degree of the polynomial for the fitting, which in turns increases the number of points we need to estimate, this hints at the fact that picking $b-a$ of constant order will be optimal.

On the other hand, it is also clear that the closer a is to 0, the more information about the value of $p'(0)$ we can infer from the interpolation. Thus, this discussion suggests that picking a as close to 0 as possible and b of constant order should give the best results. We will prove this intuition later in this section, but first will discuss robust interpolation.

Directly interpolating through the noisy data can be an unstable procedure if we do not pick the interpolating points wisely and perform a suitable regression. Recent results have shown how to perform polynomial interpolation in an essentially optimal fashion in a robust way even with a fraction of the points being outliers [12]. Let us now review the results of [12], but note that we expect that less sophisticated interpolation techniques, i.e. based on least squares regressions, should have a similar performance in practice. The reason we use the interpolation described in [12] is that it comes with very strong theoretical guarantees under minal assumptions.

We will now assume we wish to estimate a polynomial $p : [-1, 1] \to \mathbb{R}$ of degree d, as this corresponds to the setting of [12]. Note that for Hamiltonian learning, we will be interested in the case where the domain is of the form $[a, b]$ for $a, b \geq 0$. However, we can simply shift and rescale the domain to $[-1, 1]$. When we summarize our results later, we will dicuss the effect of this rescaling explicitly. We will assume we are given access to m random samples (x_i, y_i) of points such that a fraction of at least $\alpha > 1/2$ of them satisfies for some $\sigma > 0$ that:

$$p(x_i) = y_i + w_i, \quad |w_i| \leq \sigma. \tag{90}$$

There are results available for various different ways of sampling the points x_i. However, the best available sample complexity is given by sampling from the Chebyshev measure, which has density

$$\frac{1}{\pi\sqrt{1-x^2}} \tag{91}$$

on the interval $[-1, 1]$. We then have:

Theorem 5.1 (Robust polynomial interpolation)**.** *Let $p : [-1, 1] \to \mathbb{R}$ be a polynomial of degree d and assume we are given m samples (x_i, y_i) such that a fraction $\alpha > 1/2$ of them satisfies Eq.* (90) *for some $\sigma > 0$. Moreover, suppose that the x_i were sampled independently and at random from the Chebyshev measure. Then for any $\delta > 0$*

$$m = \mathcal{O}\left(d \log\left(\tfrac{d}{\delta}\right)\right) \tag{92}$$

samples suffice to with probability of success at least $1-\delta$ recover a polynomial $\hat{p}$ that satisfies:

$$\max_{x \in [-1,1]} |p(x) - \hat{p}(x)| \leq 3\sigma. \tag{93}$$

Moreover, $\hat{p}$ can be computed in time polynomial in the number of samples m.

Proof. We refer to [12, Corollary 1.5] for a proof and note that we obtain the statement by setting the parameter $\epsilon < 1/4$ in their statement. □

We note that the same result holds for random points picked from the uniform measure with $m = \mathcal{O}(d^2)$.

The result above solves our problem of robust polynomial interpolation outlined in points 1 and 2. It shows that it if we can ensure that we can approximate sufficiently many points of the polynomial up to some σ, then we also recover the whole polynomial up to some error proportional to σ. Moreover, the number of samples required only has a logarithmic overhead in d when compared with the case where we know the points exactly. As we will see later, for our puproses it will be important to choose d to be small. Thus, in a nutshell, we see that Thm. 5.1 ensures that we can reliably and robustly perform polynomial interpolation by only a small overhead when compared to when we know the points exactly.

We will later describe in more detail the algorithm given in [12] whose output satisfies the promises of Thm. 5.1. However, before that we will show how the condition in Eq. (93) ensures that we can also recover the derivative of the polynomial as long as the degree d is small.

To do that, we will resort to Markov brothers' inequality, which we restate now for completeness.

Lemma 5.1 (Markov brothers' inequality)**.** *For $d, k \in \mathbb{N}$ define the constant $C_M(d,k)$ to be given by*

$$C_M(d,k) = \frac{d^2\left(d^2-1^2\right)\left(d^2-2^2\right)\cdots\left(d^2-(k-1)^2\right)}{1\cdot 3\cdot 5\cdots(2k-1)}. \tag{94}$$

Then for any polynomial p of degree d we have that:

$$\max_{x\in[-1,1]}\left|p^{(k)}(x)\right| \leq C_M(d,k)\max_{x\in[-1,1]}|p(x)|. \tag{95}$$

Proof. We refer to [14, Theorem 1.2] for a proof and discussion of this result. □

Note that the value of $CM(d,k)$ increases exponentially with d for k constant. We remark that having further promises on the structure of the polynomial, such as the location of its zeros, can greatly improve this estimate. We once again refer to [14, Chapter 1] for a discussion on this. It would be interesting to see if recent results on the analyticity of the partition function [15] could be used in our context to also improve the error estimate. However, this general bound will suffice for our purposes.

Let us now discuss the rescaling. It is easy to see that for polynomials defined on some interval $[a,b]$, the polynomial $\tilde{p}(x) = p(\frac{b-a}{2}x+\frac{a+b}{2})$ is defined on $[-1,1]$ and we can use this simple transformation to obtain a variation of Eq. (95) for polynomials defined on general intervals. Indeed, applying Eq. (95) to $\tilde{p}$, it follows from a straightforward application of the chain rule that

$$\max_{x\in[a,b]}\left|p^{(k)}(x)\right| \leq \left|\frac{2}{(b-a)}\right|^k C_M(d,k)\max_{x\in[a,b]}|p(x)|. \tag{96}$$

From this we conclude that:

Lemma 5.2 (Extrapolating the derivative at 0)**.** *Let $p:[0,b]\to\mathbb{R}$ be a polynomial of degree d such that for some $\epsilon>0$ and $0<a<b$:*

$$\max_{x\in[a,b]}|p(x)| \leq \epsilon. \tag{97}$$

Then

$$\max_{x\in[0,a]}|p(x)| \leq \epsilon\left(\sum_{k=0}^{d}\left|\frac{2}{(b-a)}\right|^k a^k\frac{C_M(d,k)}{k!}\right) \tag{98}$$

and

$$|p'(0)| \leq \epsilon\left(\sum_{k=1}^{d}\left|\frac{2}{b-a}\right|^k a^{k-1}\frac{C_M(d,k)}{(k-1)!}\right). \tag{99}$$

Proof. It follows from Eq. (97) and Markov brothers' inequality that

$$\left|p^{(k)}(a)\right| \leq \left|\frac{2}{b-a}\right|^k C_M(d,k)\epsilon. \tag{100}$$

By a Taylor expansion we know that for $x\in[0,a]$:

$$p(x) = \sum_{k=0}^{d} p^{(k)}(a)\frac{(x-a)^k}{k!}. \tag{101}$$

The claim in Eq. (98) then follows by combining this expansion with Eq. (100) and a triangle inequality. Similarly we have

$$p'(0) = \sum_{k=1}^{d} p^{(k)}(a)(-1)^k\frac{a^{k-1}}{(k-1)!}, \tag{102}$$

for which a similar argument yields Eq. (99). □

The proposition above essentially allows us to control to what extent the derivative of a polynomial at 0 can deviate from 0 given that the polynomial is small on another interval $[a, b]$. We can then apply it to the polynomial $p - \hat{p}$, as in Eq. (93) to control the error we make by estimating the derivative at 0 by evaluating $\hat{p}(0)$.

By combining the arguments above we conclude that:

Proposition 5.1 (Precision and number of samples for robust interpolation)**.** *Let p be a polynomial of degree d. For some $0 < a < b$ define*

$$E(a,b,d) = \left(\sum_{k=1}^{d} \left| \frac{2}{b-a} \right|^k a^{k-1} \frac{C_M(d,k)}{(k-1)!} \right). \tag{103}$$

Then for $\delta > 0$ sampling

$$m = \mathcal{O}(d \log(d\delta^{-1})) \tag{104}$$

i.i.d. points (x_i, y_i) from the Chebyshev measure on $[a.b]$ satisfying

$$p(x_i) = y_i + w_i, \quad |w_i| \leq \sigma$$

for at least a fraction $\alpha > \frac{1}{2}$ of the points is sufficient to obtain a polynomial $\hat{p}$ satisfying

$$\left| p'(0) - (\hat{p})'(0) \right| \leq 3\sigma E(a,b,d). \tag{105}$$

with probability of success at least $1 - \delta$.

Proof. From Thm. 5.1 we know that this number of samples suffices to obtain the polynomial $\hat{p}$ satisfying

$$\max_{x \in [a,b]} |p(x) - \hat{p}(x)| \leq 3\sigma. \tag{106}$$

Applying Lemma 5.2 to $p - \hat{p}$ yields the claim. □

This then yields a simple condition on how small σ has to be in the regime of interest to us:

Corollary 5.1. *In the same setting as in Prop. 5.1 for some $\epsilon > 0$ let $a \leq d^{-2}$, $b = 2 + a$ and*

$$\sigma = \epsilon d^{-2}. \tag{107}$$

Then

$$|p'(0) - \hat{p}'(0)| = \mathcal{O}(\epsilon). \tag{108}$$

Proof. It is easy to see that we have:

$$C_M(d,k) \leq \frac{d^{2k}}{k!!}, \tag{109}$$

where $k!! = 1 \times 3 \times 5 \times \cdots \times (2k-1)$ is the double factorial.

Thus, we see from this and Eq. (99) that by our choice

$$a = \frac{1}{d^2}, \quad b = 2 + a \tag{110}$$

we have from Eq. (105) that the estimated polynomial $\hat{p}(0)$ satisfies

$$|\hat{p}'(0) - p'(0)| \leq 3\sigma \left(\sum_{k=1}^{d} \left| \frac{2}{(a-b)} \right|^k a^{-1} \frac{(ad^2)^k}{k!!(k-1)!} \right) \leq 3\sigma \left(\sum_{k=1}^{d} a^{-1} \frac{1}{k!!(k-1)!} \right), \tag{111}$$

where we used the fact that $a \leq d^{-2}$.

Thus, as

$$\left(\sum_{k=1}^{d} \frac{1}{k!!(k-1)!} \right) \leq e, \tag{112}$$

we conclude that with this choice of parameters we have

$$|\hat{p}'(0) - p'(0)| \leq 3e\sigma a^{-1}, \tag{113}$$

which gives the claim by our choice of σ. □

We will discuss in Sec. 6 how to specialize the discussion and results above to the scenario of Hamiltonian learning.

Supplementary Note 6: Choice of parameters and performance guarantee of the protocol

Let us now combine the results from Sections 4 and 5 to see how to pick the various parameters of the algorithm to ensure a good recovery of the couplings of the Hamiltonian.

More precisely, given a coupling parameter a_α of $\mathcal{L}_\Lambda$ we will be interested in estimating the sample complexity of obtaining an estimate $\hat{a}_\alpha$ satisfying

$$|a_\alpha - \hat{a}_\alpha| \leq \epsilon \tag{114}$$

with high probability for some given error ϵ. As extensively discussed by now, we can easily reduce estimating the couplings to estimating derivatives of time evolutions of local observables.

As expected, we will see that the main parameters we need to control are the maximal observation time $t_{\max}$ and the initial time of measurement t_0. This is showcased in the following Theorem:

Theorem 6.1 (Choice of final and initial time). *Let $\mathcal{L}_\Lambda$ be a locally bounded Lindbladian on a D-dimensional regular lattice with growth constant $g = \mathcal{O}(1)$. Let O be an observable of constant support and ρ and arbitrary quantum state. Let $\epsilon > 0$ be given. Assume that $\mathcal{L}_\Lambda$ satisfies Eq. (81) for some function h. Then picking t_0 as*

$$t_0 = \mathcal{O}\left[\left(h^{-1}\left(\frac{\epsilon}{2(e^{2.5v}-1)}\right)^D \log(\epsilon^{-1})\right)^{-2}\right] \tag{115}$$

and $t_{\max} = 2 + t_0$ and measuring the expectation value $f(t) = \operatorname{tr}\left[e^{t\mathcal{L}_\Lambda}(O)\rho\right]$ for

$$m = \tilde{\mathcal{O}}\left[\left(h^{-1}\left(\frac{\epsilon}{2(e^{2.5v}-1)}\right)\right)^D \log(\epsilon^{-1}))\right] \tag{116}$$

random times $t_i \in [t_0, t_{\max}]$ up to precision $\mathcal{O}(\epsilon)$ is sufficient to obtain an estimate $(\hat{f})'(0)$ satisfying

$$\left|(\hat{f})'(0) - \operatorname{tr}\left[\mathcal{L}_\Lambda(O)\rho\right]\right| \leq 3e\epsilon t_0^{-1} = \mathcal{O}\left[\epsilon\left(h^{-1}\left(\frac{\epsilon}{2(e^{2.5v}-1)}\right)^D \log(\epsilon^{-1})\right)^2\right]. \tag{117}$$

In particular, this estimate can be obtained from

$$\tilde{\mathcal{O}}(\epsilon^{-2}\log(\delta^{-1})) \tag{118}$$

samples from the time evolved state ρ with probability of success at least $1-\delta$.

Proof. First, note that by Lemma. 4.1, if we pick $t_{\max}$ as described above, then a polynomial p of degree

$$d = \mathcal{O}\left[\left(h^{-1}\left(\frac{\epsilon}{2(e^{2.5v}-1)}\right)\right)^D \log(\epsilon^{-1}))\right] \tag{119}$$

is sufficient to approximate the expectation value in the interval $[0, t_{\max}]$ up to an error $\epsilon/2$, assuming that ϵ is small enough to ensure that $t_{\max} \leq 2.5$. We will estimate the value of the polynomial at each point up to an error $\sigma > 0$, which is to be determined later.

Thus, by inserting the bound on the degree d in Eq. (107), we need to estimate each value of the polynomial up to a precision $\sigma = \mathcal{O}(\epsilon)$ to obtain an overall error of

$$\mathcal{O}\left[\epsilon\left(h^{-1}\left(\frac{\epsilon}{2(e^{2.5v}-1)}\right)^D \log(\epsilon^{-1})\right)^2\right] \tag{120}$$

As we imposed that the precision with which the polynomial approximates the expectation values is $\epsilon/2$, we can estimate the value of the polynomial for a given time up to an error $\mathcal{O}(\epsilon)$ from $\mathcal{O}(\epsilon^{-2})$ samples.

As we have to sample

$$\mathcal{O}(d\log(d)) = \tilde{\mathcal{O}}\left[\left(h^{-1}\left(\frac{\epsilon}{2(e^{2.5v}-1)}\right)^D \log(\epsilon^{-1})\right)\right] \tag{121}$$

points to perform the stable interpolation, we obtain the advertised sample complexity. □

	Sample Complexity	Number of points	Initial time
Finite range	ϵ^{-2}	polylog(ϵ^{-1})	polylog(ϵ)
Exponentially	ϵ^{-2}	polylog(ϵ^{-1})	polylog(ϵ)
Algebraically ($\alpha \geq 5D-1$)	$\epsilon^{-2\frac{\alpha-3D}{\alpha-5D}}$	$\epsilon^{-\frac{D}{\alpha-5D}}$	$\epsilon^{\frac{2D}{\alpha-5D}}$

Supplementary Table VI. Scaling of different resources required to obtain a recovery up to additive error ϵ of a parameters of the evolution. We have only included the leading order term and α denotes the decay of the potential in space, whereas D the dimension of the lattice.

For the case of strictly local or exponentially decaying interactions we have that

$$h^{-1}\left(\frac{\epsilon}{2(e^{2.5v}-1)}\right) = \text{poly}\,\log(\epsilon^{-1}). \tag{122}$$

In that case the sample complexity is of order $\tilde{\mathcal{O}}(\epsilon^{-2})$. Thus, in this case we see that the inverse initial time t_0^{-1} and the number of points we need to sample from is polylogarithmic in ϵ. Furthermore, the sample complexity to obtain an error ϵ is also $\tilde{\mathcal{O}}(\epsilon^{-2})$ up to polylogarithmic corrections.

For the sake of completeness, let us now discuss the conditions under which our protocol works beyond the setting of exponentially decaying or short-range interactions. From Eq. (117) the condition for our procedure to work becomes transparent: we need that

$$\left(h^{-1}\left(\frac{\epsilon}{2(e^{2.5v}-1)}\right)^{D}\log(\epsilon^{-1})\right)^{-2} = o(\epsilon^{-1}). \tag{123}$$

Indeed, in this case we have the property that it is possible to suitably re-scale the error ϵ to ensure that the total precision is at some desired precision $\tilde{\epsilon}$. For instance, let us assume that

$$h^{-1}\left(\frac{\epsilon}{2(e^{2.5v}-1)}\right) = \mathcal{O}(\epsilon^{-r}), \tag{124}$$

for some $r > 0$. As we discuss later, this is typically the case for algebraically decaying interactions. For such a LR-bound, we see that the resulting error in Eq. (117) is

$$\mathcal{O}(\epsilon^{1-2Dr}\log(\epsilon^{-1})^2). \tag{125}$$

Ignoring the $\log(\epsilon^{-1})$ term, we see that by picking $\epsilon = \tilde{\epsilon}^{\frac{1}{1-2Dr}}$ we can ensure an error of order $\tilde{\epsilon}$ for the estimate. Thus, the growth of h^{-1} has to be at most $r \leq \frac{1}{2D}$ and the sample complexity would also grow like $\tilde{\mathcal{O}}(\epsilon^{-2-2Dr-r})$, as we would need to sample $\tilde{\mathcal{O}}(d) = \tilde{\mathcal{O}}(\epsilon^{-r})$ points up to precision $\tilde{\epsilon}^{\frac{1}{1-2Dr}}$.

Thus, we see that our protocol has a sample complexity that is independent of the system size to estimate one parameter and the expected ϵ^{-2} scaling for short range evolutions evolutions, up to log factors. For algebraically decaying interactions, however, the sample complexity has a worse scaling that depends on the exact decay of the potential, but still independent of system size.

We summarize the sample complexities, smallest initial time t_0 and number of different times steps we need for various different potentials in Table VI.

A. Algorithm for robust polynomial interpolation

Now that we have established that the results of [12] indeed allow us to estimate the derivative at 0, let us now describe their polynomial interpolation algorithm in more detail for completeness. The algorithm consists of two parts, one ℓ_1 regression and an iteration of ℓ_∞ regressions. Following [12], we will only consider the case in which we interpolate over $[-1,1]$. But it is straightforward to also interpolate over other intervals by a suitable affine transformation of the domain, as discussed before.

a. ℓ_1 regression: before we define the ℓ_1 regression, we need to define the Chebyshev partitions:

Definition 6.1 (Chebyshev partitions)**.** *Let $m \in \mathbb{N}$ be given. The size m Chebyshev partitions of* $[-1,1]$ *is the set of intervals* $I_j = \left[\cos\frac{\pi j}{m}, \cos\frac{\pi(j-1)}{m}\right]$ *for* $1 \leq j \leq m$.

We also define $\mathcal{P}_d$ to be the space of polynomials of degree at most d.

With these definitions at hand, we define the ℓ_1 regression solution as follows:

Definition 6.2. *Given a set of n points (x_i, y_i) and $m \in \mathbb{N}$, we define the result of the degree d ℓ_1 regression with m Chebyshev partitions $\hat{p}$ to be the polynomial*

$$\text{argmin}_{\hat{p}\in\mathcal{P}_d} \sum_{i=1}^{n} |I_j| \,\text{mean}_{x_i\in I_j} |y_i - \hat{p}(x_i)| \,, \tag{126}$$

where $\mathcal{P}_d$ is the set of polynomials of degree d.

Note that the optimization problem above is a linear program and, thus, can be solved efficiently. Solving the ℓ_1 regression problem with $n = \mathcal{O}(d\log(d))$ samples from the Chebyshev measure is guaranteed to give us a good solution on average. More precisely, as shown in [12, Lemma 1.2], the solution is guaranteed to satisfy

$$\|p - \hat{p}\|_{\ell_1} = \mathcal{O}(\sigma), \tag{127}$$

where as usual σ is the error in each estimate y_i and

$$\|p - \hat{p}\|_{\ell_1} = \int_{-1}^{1} |p(x) - \hat{p}(x)| \,dx. \tag{128}$$

However, the results of the previous sections required us to obtain a good solution in the $\|\cdot\|_{\ell_\infty}$ distance, and in general

$$\|p - \hat{p}\|_{\ell_1} = \mathcal{O}(d^2\|p - \hat{p}\|_\infty). \tag{129}$$

Although, as commented in the last section, we are interested in the regime of polynomial of relatively small degree, by adding a ℓ_∞ regression iteration on top of the ℓ_1 regression, it is possible to get rid of this d^2 prefactor.

b. ℓ_∞ regression: besides getting rid of the unwanted d^2 factor on the promise for the error of the ℓ_1 regression, adding a ℓ_∞ regression step also has the favourable feature of making the whole procedure more robust to outliers in the data.

Definition 6.3 (ℓ_∞ regression)**.** *Given a set of n points (x_i, y_i) and $m \in \mathbb{N}$ given. For the m Chebyshev partitions I_j, choose $\tilde{x}_j \in I_j$ arbitrarily and let*

$$\widetilde{y}_j = \text{median}_{x_i\in I_j}\, y_i. \tag{130}$$

We define the result of the degree d ℓ_∞ regression with m Chebyshev partitions $\hat{p}$ to be the polynomial $\hat{p} \in \mathcal{P}_d$

$$\text{argmin}_{\hat{p}\in\mathcal{P}_d} \max_{j\in[m]} |\widehat{p}\,(\widetilde{x}_j) - \widetilde{y}_j| \,. \tag{131}$$

Note that the problem in Eq. (131) also corresponds to a linear program and, thus, can be solved efficiently. The output of the ℓ_∞ regression algorithm is guaranteed to satisfy

$$\|\hat{p} - p\|_\infty \le 2.5\sigma + \frac{1}{2}\|p\|_\infty \tag{132}$$

as long as $m = \mathcal{O}(d)$ and the we pick $n = \mathcal{O}(d\log(d))$ samples from the Chebyshev measure, as shown in [12, Lemma 1.3]. Thus, the procedure gives us a promise of recovery in ∞-norm up to the unwanted $\|p\|_\infty$ term. This can be solved by iterating the ℓ_∞ regression step.

c. Iterating the ℓ_∞ step: the last step to obtain the desired robust polynomial interpolation is to iteratively apply the ℓ_∞ iteration step to the residual. More precisely, we first perform the ℓ_1-regression on our data, obtaining a polynomial $\hat{p}_0$. We can then define the new data points

$$(x_i, \tilde{y}_i^0 = y_i - \hat{p}_0(x_i)) \tag{133}$$

and run the ℓ_∞ interpolation on this residual error, obtaining a polynomial $\hat{p}_1$. From Eq. (129) and our promise on the output of the ℓ_∞ interpolation, we know that the result of the interpolation will satisfy

$$\|p - \hat{p}_1\|_\infty \le 2.5\sigma + \frac{1}{2}\mathcal{O}(d^2\sigma). \tag{134}$$

But then we can iterate this procedure by just running the ℓ_∞ regression on

$$(x_i, \tilde{y}_i^1 = y_i - \hat{p}_1(x_i)). \tag{135}$$

Each time we run the interpolation on the residual, we exponentially reduce the error. By repeating the procedure $\mathcal{O}(\log_2(d))$ times, we then arrive at a polynomial satisfying the promises of Thm. 5.1.

Note, however, that the procedure used in Sec. III of the main text to demonstrate the viability of our method differs slightly from the ones discussed here. The main difference is that we used equally spaced time steps that were not random. However, in spite of this difference, we still obtained high quality solutions.

B. Certifying the degree of the polynomial in the fitting

Our approach requires us to fit the expectation values to a low-degree polynomial. However, it is natural to ask how to pick the degree of the polynomial we fit the data to and to certify that the degree was chosen correctly. A heuristic approach to this problem worked remarkably well in our numerics: we increased the degree d until the maximal difference between the interpolating polynomial we obtained and the data we collected would fall below $\mathcal{O}(\epsilon)$, the target accuracy.

In Sec. 7 A we give a version of our polynomial approximation results with explicit constants. The exact required degree is given in Eq. (148). However, our numerics suggests that a polynomial of degree orders of magnitude smaller than given by the bound suffices to obtain a good approximation and offers greater numerical stability. We leave to future work to obtain more refined estimated on the degree required.

Supplementary Note 7: Lieb-Robinson bounds

This section gives a brief overview of Lieb-Robinson bounds. In particular, we give more explicit formulas for the functions h (see Formula (13) in the main text) in terms of the decay of the interactions and the dimension of the lattice. Lieb-Robinson bounds are by now a standard tool in quantum many-body systems and quantum information theory and we refer to [8, 9, 11, 16–20] for a more general overview over the mathematical background and some latest bounds for algebraically decaying interactions.

At the heart of any Lieb-Robinson bound is the intuitive idea that if interactions in a system happen locally this should imply a bound on how fast information can be transmitted. The usual way to codify this property for Hamiltonian systems in the Heisenberg picture is to give a bound on the operator norm of the commutator between a time-evolved observable $O_Y(t)$ initially located in region Y and a second observable A_X located in a region X in the distance $\mathrm{dist}(X,Y)$ between the regions X, Y, i.e. a bound of the form

$$\|[A_X, O_Y(t)]\| \leq Ch(\mathrm{dist}(X,Y))(e^{vt} - 1), \tag{136}$$

where C will typically depend on the size of the regions $|X|$ and $|Y|$ as well as on the operator norms of A and $O(t)$. However, in the context of Markovian dynamics and master equations, the bound is usually generalized by substituting the super-operator $[A_X, \cdot]$ for an arbitrary bounded super-operator $\mathcal{K}_X : \mathcal{M}_{2^n} \to \mathcal{M}_{2^n}$ supported on X leading to a Lieb-Robinson-bound of the form

$$\|\mathcal{K}_X(O_Y(t))\| \leq Ch(\mathrm{dist}(X,Y))(e^{vt} - 1), \tag{137}$$

with C depending on $\|\mathcal{K}_X\|_{\infty\to\infty,cb}$.However, if $\mathcal{K}_X$ is of the form $\mathcal{K}_X = [A_X, \cdot]$, we have $\|\mathcal{K}_X\|_{\infty\to\infty,cb} \leq 2\,\|A_X\|_\infty$, which allows us to recover the commutator [10, 19]. In the following, we consider a regular lattice Λ and assume that the dynamics is generated by a Lindbladian that decomposes according to

$$\mathcal{L} = \sum_{X\subset\Lambda} \mathcal{L}_X\ . \tag{138}$$

Following [9, 19, 21], we define the maximal interaction strength

$$J = \sup_{X\subset\Lambda} \|\mathcal{L}_X\|_{1\to1,cb} \tag{139}$$

as well as the decay behaviour of the interactions

$$\mu(r) = \sup_{X\subset\Lambda:\mathrm{diam}(X)=r} \frac{\|\mathcal{L}_X\|_{1\to1,cb}}{J} \tag{140}$$

in terms of the stabilized 1-to-1-norm $\|T\|_{1\to1,cb} = \sup_n \|T \otimes \mathrm{id}_n\|_{1\to1}$. We can then characterize $\mathcal{L}$ as finite range if $\mu(r) = 0$ for $r \geq R > 0$, exponentially decaying if $\mu(r) \leq e^{-\mu r}$ and algebraically decaying if $\mu(r) \leq (1+r)^{-\alpha}$ for $\alpha > 0$ and state the following Lieb-Robinson-bound for Lindbladians:

Theorem 7.1 (dissipative LR-bound [19]). *Let $\mathcal{L}$ be a Lindbladian of the form* (138)*, O_Y an observable supported on $Y \subset \Lambda$ and $\mathcal{K}_X : \mathcal{M}_{2^n} \to \mathcal{M}_{2^n}$ with $\mathcal{K}_X(id_X) = 0$. Then*

$$\|\mathcal{K}_X(O_Y(t))\| \leq \|\mathcal{K}_X\|_{\infty\to\infty,cb} \|O_Y\| \min(|X|,|Y|) h(\mathrm{dist}(X,Y))(e^{vt}-1), \tag{141}$$

with $h(r) = e^{-\nu r}$ for $\mathcal{L}$ exponentially decaying or finite range and $h(r) = (1+r)^{\nu}$ if $\mathcal{L}$ is algebraically decaying with $\alpha > 2D+1$ with $\nu < \alpha - (2D+1)$, where D is the dimension of the lattice.

As stated above, in this work, we require a slightly different formulation of the LR-bound as given in Formula (7) of the main text, namely

$$\|(e^{t\mathcal{L}_\Lambda} - e^{t\mathcal{L}_{\Lambda_{r(Y)}}})(O_Y)\| \leq c_1 h(\mathrm{diam}(\Lambda_{r(Y)}))(e^{vt}-1), \tag{142}$$

which reflects directly that the dynamics of the system can already be described by a generator $\mathcal{L}_{\Lambda_{r(Y)}}$ restricted to a region $\Lambda_{r(Y)}$ of diameter r around the initial support Y of the observable O_Y. To convert a bound of the form (137), we follow the reasoning given in [10, 19].

We can express the difference of the dynamics generated by the full Lindblad generator $\mathcal{L}_\Lambda$ as compared to a restriction $\mathcal{L}_{\Lambda_r}$ to the subset $\Lambda_r \subset \Lambda$ according to

$$(e^{t\mathcal{L}_\Lambda} - e^{t\mathcal{L}_{\Lambda_r}})O_Y = -\int_0^t \mathrm{d}s\, \partial_s \left(e^{s\mathcal{L}_{\Lambda_r}} e^{(t-s)\mathcal{L}_\Lambda}\right) O_Y = \int_0^t \mathrm{d}s\, e^{s\mathcal{L}_{\Lambda_r}} \left(\mathcal{L}_\Lambda - \mathcal{L}_{\Lambda_r}\right) e^{(t-s)\mathcal{L}_\Lambda}(O_Y) \tag{143}$$

Taking norms on both sides, we therefore obtain an upper bound of the form

$$\left\|(e^{t\mathcal{L}_\Lambda} - e^{t\mathcal{L}_{\Lambda_r}})O_Y\right\| \leq \sum_{X \not\subset \Lambda_r} \int_0^t \mathrm{d}s \left\|\mathcal{L}_X e^{(t-s)\mathcal{L}_\Lambda}(O_Y)\right\| . \tag{144}$$

We notice, that the term inside the integral is exactly of the form of the left-hand side of (137) with $\mathcal{K}_X = \mathcal{L}_X$. Hence, we can insert the standard LR-bound for dissipative dynamics from (137) here and are left with a combinatorial problem to obtain a bound on the approximation. This can be done explicitly for several standard interaction decays, such as finite range, exponentially decaying or algebraically decaying interactions [10, 19]. In particular, based on the Lieb-Robinson bound in Thm. 7.1, we obtain

Lemma 7.1 ([19]). *Let $\mathcal{L}$ be a Lindbladian of the form* (138)*, O_Y an observable supported on $Y \subset \Lambda$ and $r > 0$ hold. Then for $\mathcal{L}_{\Lambda_r(y)} = \sum_{X \subset \Lambda_r(y)} \Lambda_X$ we have*

$$\left\|\left(e^{t\mathcal{L}} - e^{t\mathcal{L}_{\Lambda_r(y)}}\right) O_Y\right\| \leq \|O_Y\|\, |Y| J \frac{e^{vt} - 1 - vt}{v} h(r) \tag{145}$$

with $h(r)$ exponentially decaying in r for $\mathcal{L}$ finite range or exponentially decaying and $h(r)$ decaying as $(1+r)^{-\beta}$ if $\mathcal{L}$ is algebraically decaying with $\alpha > 2D+1$ and $\beta = \alpha - 3D$ for $\alpha \geq 5D-1$ and $\beta = \frac{1}{2}(\alpha - D - 1)$ if $\alpha \leq 5D-1$, where again D is the dimension of the lattice.

We remark that all these bound give us the required independence of the right-hand side from the overall system size. We expect that with the help of recent more stringent estimates on Hamiltonians with algebraic decay, it will most likely be possible to extend and strengthen these bounds for other algebraic decays.

A. Explict Lieb-Robinson velocities

As discussed in section 10, making a priori estimates about the sampling complexity beyond the principle scaling, requires explicit knowledge about the constants in the LR-bounds we are using, in order to obtain a bound on the degree of the involved polynomials. This includes in particular the Lieb-Robinson velocity that governs the decay behaviour of the equations in time. Hence, within this section, we will discuss some models were this dependence can be made more explicit.

First of all, we note that for a given anticipated interaction graph and assuming a bound on the interaction strength and decay behaviour as defined in (139) and (140), according to [19] the Lieb-Robinson velocity can always be chosen according to

$$2J \sup_{x,y\Lambda} \sum_{n\geq 0} \frac{|b_x(n)|}{|b_x(n-1)|} \sum_{r\geq n} \mu(r)|b_y(r)|h(r) \leq v, \tag{146}$$

where $b_x(n)$ denotes the ball of size n around lattice side x and the function $h(r)$ is specified in Thm. 7.1 depending on the decay of the interactions. This will in principle allow us to obtain an explicit bound for any anticipated model our protocol can be applied to.

However, more explicit bounds can be derived in the case of stronger assumptions on the locality of the interactions. Indeed, let us consider the case of finite-range interactions specified on an interaction graph with (hyper)edge set E that satisfies the following requirements [11]

1. finite interaction strength: $g = sup_{X\in E} \|\mathcal{L}_{\mathcal{X}}\| \leq \infty$
2. finite neighbourhood: $Z = \max_{X\in E} |\{Y \in E : X \cap Y \neq \emptyset\} \leq \infty$
3. bounded growth of neighbours: $\max_{X\in E} |\{Y : \mathrm{dist}(X,Y) = n\}| \leq Mn^{\eta}$

For such a systems, [11, Thm.2] derives a Lieb-Robinson bound according to Lemma 7.1 with the right-hand side of (145) given by

$$\frac{2M}{Z} \|O_Y\| \, r^{\eta} e^{(egZ)\cdot t - r} = \frac{2M}{Z} e^{v\cdot t} h(r) \tag{147}$$

with $h(r) = r^{\eta}e^{-r}$ and the LR-velocity $v = egZ$. Hence, following the reasoning in section 4, we choose r as $h^{-1}(\frac{Z\varepsilon}{4M\|O_Y\| \exp(v\cdot t_{max})})$, which for$\|O_Y\| \leq 1$ and for r large enough such that $(1-\eta\frac{\log r}{r}) > \frac{2}{3}$ means that r should be chosen as $\frac{3}{2}\log\left(\frac{4Me^{(egZ)\cdot t_{max}}}{Z\varepsilon}\right)$. This in turn results for the truncated Taylor-expansion for the corresponding generator $\mathcal{L}_{\Lambda_y(r)}$ restricted to the region $\Lambda_y(r)$ in a polynomial degree of $d = 2et_{max}g|\Lambda_y(r)| - 1$, which in the case of a D-dimensional lattice results in

$$d_{exp} = 2et_{max}g\left(\frac{3}{2}\log\left(\frac{4Me^{(egZ)\cdot t_{max}}}{Z\varepsilon}\right) + \mathrm{diam}(\mathrm{supp}(O_Y))\right)^D \log(\varepsilon^{-1}) - 1\ . \tag{148}$$

Following the reasoning in section 6, this implies a required minimal starting time $t_0 = d_{exp}^2$ and maximal evolution time $t_{max} = 2 + t_0$ as well as sampling $m = \mathcal{O}\left(d\log(d\delta^{-1}\right)$ evolution times within this interval according to the Chebychev distribution in order to succeed with the polynomial interpolation.

Finally, let us mention that by assuming even more structure on the interactions and using the method developed in [22] based on the so-called commutativity graph, even stronger bounds on the LR-velocity can be obtained for certain models. Indeed, for example for the translation invariant transverse field Ising model in two-dimensions, [22] shows that $v \sim \sqrt{g}$ instead of the linear dependence discussed before and we would expect that this scaling will also be an upper bound in the case of non-translation invariance.

Supplementary Note 8: Parallelizing the Measurements: shadow process tomography

In this section we introduce a method to parallelize the estimation of the Pauli overlaps required for our protocol. For a quantum channel on n qubits Φ and two collections of Pauli strings $P_a^1, \ldots, P_a^{K_1}$ and $P_b^1, \ldots, P_b^{K_2}$ that have combined weight (i.e. number of Paulis that are nonidentity) $\omega_a + \omega_b$ at most ω it allows us to estimate all the overlaps of the form

$$2^{-n}\,\mathrm{tr}\left[P_a^j \Phi(P_b^k)\right] \tag{149}$$

up to an error ϵ with probability at least $1-\delta$ from a number of samples that grows like $\mathcal{O}(3^w \log(K_1K_2\delta^{-1})\epsilon^{-2})$. Moreover, it only requires us to prepare simple Pauli eigenstates and measure in Pauli eigenbases. Recently two works [23, 24] have considered how to generalize the shadows protocol to the setting of process tomography. Here we give an alternative analysis that has a better sample complexity than the previous works when specialized to Pauli strings. Thus, we believe that this section may be of independent interest.

The protocol we will introduce now allows us to estimate all the data required for our Hamiltonian learning protocol in parallel. Every round i of the protocol is performed as follows:

1. Draw two Pauli strings $B_i, S_i \in \{\sigma_x, \sigma_y, \sigma_z\}^n$ uniformly at random and also a sign vector $E_i = \{-1, +1\}^n$ uniformly at random.

2. Prepare the quantum state $\rho_i = \otimes_{j=1}^n \phi_j(S_i, E_i)$, where $\phi_j(S_i, E_i)$ is an eigenstate of the $j-$th Pauli on the string S_i corresponding to the eigenvalue in the j-th entry of E_i.

3. Evolve ρ_i by Φ.

4. Measure in the Pauli basis defined by B_i. Denote the measurement outcome by M_i.

5. Output (B_i, S_i, E_i, M_i).

Let us now introduce some notation to explain how to postprocess the samples obtained from the protocol above.

Let P_a and P_b be the given Pauli operators on n qubits. We say that a basis B_i overlaps with P_a if all qubits on which P_a acts non trivially are also measured in the same basis. For example, $P_a = \sigma_x \otimes \sigma_y \otimes I$ and $B_i = \{\sigma_x, \sigma_y, \sigma_z\}$ overlap. However, $P_a = \sigma_x \otimes \sigma_y \otimes I$ and $B_i = \{\sigma_x, \sigma_z, \sigma_z\}$ do not overlap. Moreover, if the basis and P_a overlap, we say that the measurement outcome M_i overlaps positively if we measure a positive eigenstate of P_a. Otherwise, we say it is negative.

We will also define a similar notion of the state overlap given the Pauli P_b, the basis S_i and sign E_i. We say that P_b and (S_i, E_i) overlap positively if S_i coincides with P_b on all qubits it acts non trivially and the state ρ_i is a positive eigenstate of P_b. We say we overlap negatively if it is a negative eigenstate. We will also define $\omega(P_a)$ to be the number of qubits on which P_a acts non trivially.

We can now finally introduce a random variable given the Paulis P_a, P_b and the data from the experiment B_i, S_i, E_i and M_i. Let us define a function in terms of the outcomes and inputs of one round of the protocol:

$$X_{a,b}(B_i, S_i, E_i, M_i) = \begin{cases} 0 & \text{if } B_i \text{ does not overlap with } P_a \text{ or } S_i \text{ does not overlap with } P_b, \\ 3^{\omega(P_a)+\omega(P_b)}/2 & \text{if } (B_i, S_i) \text{ overlap with } (P_a, P_b), \text{ and both do so positively}, \\ 3^{\omega(P_a)+\omega(P_b)}/2 & \text{if } (B_i, S_i) \text{ overlap with } (P_a, P_b), \text{ and both do so negatively}, \\ -3^{\omega(P_a)+\omega(P_b)}/2 & \text{if } (B_i, S_i) \text{ overlap with } (P_a, P_b), \text{ one positively, the other negatively}. \end{cases} \tag{150}$$

We will now show that:

$$\mathbb{E}(X_{a,b}) = 2^{-n} \operatorname{tr}(P_a \Phi(P_b)). \tag{151}$$

and

$$\mathbb{E}(X_{a,b}^2) \leq 3^{w(P_a)+w(P_b)} \tag{152}$$

Before we prove that, let us discuss how these estimates on moments on $X_{a,b}$ suffice to obtain the claimed sample complexity. As in other works on classical shadows, it will be crucial to use the method of median of means estimator [25] to estimate the expectation value of $X_{a,b}$. The method of medians of means works as follows. We take a sample of size S and divide it into K subsets of size B, i.e. $S = KB$. We then compute the empirical mean on each of the K subsamples. Denote them by $\hat{\mu}_i$, with $1 \leq i \leq K$. We then set our estimator of the mean to be:

$$\hat{\mu}_{\text{MoM}} = \text{median}(\hat{\mu}_1, \ldots, \hat{\mu}_K). \tag{153}$$

The main property of this estimator is that we have that if the variance of $X_{a,b}$ is σ^2, then:

$$\mathbb{P}(|\hat{\mu}_{\text{MoM}} - \mathbb{E}(X_{a,b})| \geq \epsilon) \leq e^{-2K\left(\frac{1}{2} - \frac{\sigma^2}{B\epsilon^2}\right)}. \tag{154}$$

In particular, if we pick $B = 4\sigma^2\epsilon^{-2}$ and $K = 2\log(\delta^{-1})$, then:

$$\mathbb{P}(|\hat{\mu}_{\text{MoM}} - \mathbb{E}(X_{a,b})| \geq \epsilon) \leq \delta. \tag{155}$$

We see that the median of means method allows to have a logarithmic scaling of the error probability from a bound on the variance. On the other hand, just using the empirical mean directly combined with an estimate on the variance gives a polynomial dependence only.

Armed with these facts about the median of means estimator, the following result immediately follows from (151) and (152):

Corollary 8.1. *Let $\{P_a^l\}_{l=1}^{K_1}$ and $\{P_b^j\}_{j=1}^{K_2}$ be two collections of Pauli matrices on n qubits such that for any l, j the following condition*

$$\omega(P_a^l) + \omega(P_b^j) \leq \omega, \tag{156}$$

holds. Then

$$O(3^\omega \log{(K_1 K_2 \delta^{-1})} \epsilon^{-2}) \tag{157}$$

runs of the protocol above suffice to obtain an estimate $e_{a,b}^{l,j}$ satisfying

$$\left| |2^{-n} \operatorname{tr}\left[P_b^l \Phi(P_a^j)\right] - e_{a,b}^{l,j} \right| \leq \epsilon \tag{158}$$

for all pairs l, j with probability at least $1 - \delta$.

Proof. Let $X_{a,b}^{l,j}$ be the random variable we have defined above for a pair of Paulis P_a^l and P_b^j. By the bound in (152) we have that $\mathbb{E}((X_{a,b}^{l,j})^2) \leq 3^\omega$, holds. Thus, for the median of means estimator with $B = 3^\omega \epsilon^{-2}$ samples per group and $K = 2\log(\delta^{-1} K_1 K_2)$ we have an estimate satisfying (158) with probability of failure at most $\delta/K_1 K_2$.

By a union bound, the median of means estimator of all $K_1 K_2$ combinations of P_a^l and P_b^j satisfy (158) with probability of failure at most δ. The total number of samples required for this is

$$S = KB = O(3^\omega \log{(K_1 K_2 \delta^{-1})} \epsilon^{-2}), \tag{159}$$

which yields the claim. □

To conclude we only need to show that (151) and (152) hold. Let us start with the expectation value. First, note that

$$\mathbb{E}(X_{a,b}) = \mathbb{E}(X_{a,b} | B \text{ and } S \text{ overlap}) \mathbb{P}(B \text{ and } S \text{ overlap}), \tag{160}$$

holds. Here we say that B and S overlap if B overlaps with P_a and S overlaps with P_b. The latter formula holds, because if we do not have overlap then $X_{a,b} = 0$ by the case 1 of the definition (150). Now observe that

$$\mathbb{P}(B \text{ and } S \text{ overlap}) = 3^{-\omega(P_a)} 3^{-\omega(P_b)}. \tag{161}$$

This holds because we have a $1/3$ chance of "hitting the right Pauli" at each point of the support of either P_a or P_b and they are all independent. Thus, all we need is to determine

$$\mathbb{E}(X_{a,b} | B \text{ and } S \text{ overlap}). \tag{162}$$

Here we distinguish four cases:

1. S overlaps positively with P_b and B overlaps positively with P_a.
2. S overlaps negatively with P_b and B overlaps negatively with P_a,
3. S overlaps positively with P_b and B overlaps negatively with P_a,
4. S overlaps negatively with P_b and B overlaps positively with P_a.

We can then break down the expectation of (160) down into the cases:

$$\mathbb{E}(X_{a,b} | B \text{ and } S \text{ overlap}) \mathbb{P}(B \text{ and } S \text{ overlap}) = \sum_{i=1}^{4} \mathbb{E}(X_{a,b} | B \text{ and } S \text{ overlap}, c = i) \mathbb{P}(c = i), \tag{163}$$

where c is a random variable which keeps track of which case we have. By definition, if $c = 1, 2$, then

$$\mathbb{E}(X_{a,b} | B \text{ and } S \text{ overlap}, c = 1, 2) = 3^{\omega(P_a) + \omega(P_b)}/2. \tag{164}$$

If $c = 3, 4$:

$$\mathbb{E}(X_{a,b} | B \text{ and } S \text{ overlap}, c = 3, 4) = -3^{\omega(P_a) + \omega(P_b)}/2. \tag{165}$$

Note that the $3^{\omega(P_a)+\omega(P_b)}$ counteracts the $\mathbb{P}(B \text{ and } S \text{ overlap})$ term, up to an additional 1/2 factor.

Thus, all that is left is to estimate

$$\mathbb{P}(c=i), \quad i=1,2,3,4. \tag{166}$$

Let us estimate $\mathbb{P}(c=1)$, the other cases will be analogous. To this end, we will introduce Q_a^+ and Q_a^-, which are the projectors onto the positive and negative eigenvalues, respectively, of the support of P_a. We will use analogous notation for Q_b^+ and Q_b^-. Clearly, $P_a = (Q_a^+ - Q_a^-) \otimes I^{\otimes n-\omega(P_a)}$, holds. Here and in what follows we will always assume for simplicity that the Pauli strings are supported on the first qubits.

Let us estimate the expected initial state for the case $c=1$. Conditioned on being a state with positive overlap with P_b, by construction we know that the state is uniformly distributed on the positive eigenspace of P_b. This corresponds to the state

$$\sigma = \frac{Q_b^+}{2^{\omega(P_b)-1}} \otimes \left(\frac{\mathbb{I}}{2}\right)^{\otimes n-\omega(P_b)}. \tag{167}$$

Given that this is the initial state and that we are measuring in the eigenbasis of P_a (recall that we have overlap), the probability of measuring a positive outcome is $\mathrm{tr}\,(Q_a^+ \otimes \mathbb{I}^{n-\omega(P_a)}\Phi(\sigma))$.

We conclude that

$$\mathbb{P}(c=1) = \frac{1}{2}\,\mathrm{tr}\,(Q_a^+ \otimes \mathbb{I}^{n-\omega(P_a)}\Phi(\sigma)) = \frac{1}{2^{n-1}}\,\mathrm{tr}\,(Q_a^+ \otimes \mathbb{I}^{n-\omega(P_a)}\Phi(Q_b^+ \otimes \mathbb{I}^{n-\omega(P_b)})). \tag{168}$$

Similarly

$$\mathbb{P}(c=2) = \frac{1}{2^{n-1}}\,\mathrm{tr}\,(Q_a^- \otimes 1^{n-\omega(P_a)}\Phi(Q_b^- \otimes 1^{n-\omega(P_b)})), \tag{169}$$

$$\mathbb{P}(c=3) = \frac{1}{2^{n-1}}\,\mathrm{tr}\,(Q_a^+ \otimes 1^{n-\omega(P_a)}\Phi(Q_b^- \otimes 1^{n-\omega(P_b)})), \tag{170}$$

$$\mathbb{P}(c=4) = \frac{1}{2^{n-1}}\,\mathrm{tr}\,(Q_a^- \otimes 1^{n-\omega(P_a)}\Phi(Q_b^+ \otimes 1^{n-\omega(P_b)})), \tag{171}$$

As

$$2^{-n}\,\mathrm{tr}\,(P_a\Phi(P_b)) = 2^{-n}(\mathrm{tr}\,(Q_a^+\Phi(Q_b^+)) + \mathrm{tr}\,(Q_a^-\Phi(Q_b^-)) - \mathrm{tr}\,(Q_a^+\Phi(Q_b^-)) - \mathrm{tr}\,(Q_a^-\Phi(Q_b^+))), \tag{172}$$

we conclude that

$$\mathbb{E}(X_{a,b}) = 2^{-n}\,\mathrm{tr}\,(P_a\Phi(P_b)). \tag{173}$$

Computing the second moment of $X_{a,b}$ turns out to be quite simple. Note that the only nonzero value the random variable $X^2_{(a,b)}$ takes is $3^{2\omega(P_a)+2\omega(P_b)}/4$ with probability $3^{-\omega(P_a)-\omega(P_b)}$. Thus, we clearly have that:

$$\mathbb{E}(X^2_{(a,b)}) = 3^{\omega(P_a)+\omega(P_b)}/4, \tag{174}$$

which yields (152). This concludes all the computations required for Cor. (8.1).

Note that the protocol only requires the preparation of Pauli states and Pauli measurements. Thus, it should be feasible to implement it on near term devices. Additionally, the required postprocessing is efficient, as evaluating the value of $X_{a,b}$ can be efficiently done given a sample.

Furthermore, note that if we further have the information that we do not to wish to recover certain bases (i.e. we do not wish to recover Pauli strings with Y terms), it is possible to adapt the protocol and do not prepare initial states or measure in that basis. This will reduce the sample complexity accordingly.

Supplementary Note 9: Robustness of the protocol to various errors

It is natural to inquire to what extent our protocol is robust against state preparation and measurement errors (SPAM) and to what extent it still outputs reliable estimates whenever the underlying assumptions are only valid approximately. The goal of this subsection is to address all of these concerns and show that our protocol is highly robust.

In the following subsections we will discuss the following forms of robustness:

1. perturbations of the locality of the interaction.
2. SPAM errors.
3. unknown structure of interactions.

By unknown structure of interactions we mean the scenario where we do not know exactly which qubits are actually coupled to each other, i.e. the precise form of the generator is not given, but we assume such a local structure exists. And by approximately local generators we mean the case of Lindbladians that are of the form $\mathcal{L} = \mathcal{L}' + \Delta$, where $\mathcal{L}$ is a Lindbladian that satisfies our locality assumptions and Δ is an arbitrary perturbation. Note that although we could extend all of our results to dynamics that have k-body interactions, we will here restrict to at most $2-$body interactions for simplicity.

But before discussing more sophisticated bounds, let us make the general observation that the polynomial interpolation method we are using is robust to noise in the data. This is both in the sense of there being outliers in the data and the estimates we obtain for the points being close to that of a polynomial.

Thus, we have the following general robustness statement:

Lemma 9.1. *Let $\mathcal{L}$ be a Lindbladian on a D-dimensional regular lattice and $\epsilon > 0$ be given. Suppose we can measure the expectation value of two-body Pauli observables in the time interval $[t_0, t_{\max}]$ under a family of quantum channels $\{\Phi_t\}_{t_0 \le t \le t_{\max}}$ s.t. for all $t \in [t_0, t_{\max}]$ we have for $2-$body Paulis P:*

$$\|(\Phi_t^* - e^{t\mathcal{L}})(P)\| \le \mathcal{O}(\epsilon/\log(\epsilon^{-1})). \tag{175}$$

for

$$t_0^{-1} = \mathcal{O}\left[\mathrm{polylog}(\epsilon^{-1})\right] \tag{176}$$

and $t_{\max} = 2 + t_0$. Assume further that we can prepare Pauli eigenstates and perform Pauli measurements up to an error $\mathcal{O}(\epsilon/\log(\epsilon^{-1}))$. Then, measuring such Pauli measurements on Pauli eigenstates under the evolution of Φ_t^* for

$$m = \mathcal{O}\Big[\,\mathrm{polylog}(\epsilon^{-1})\Big] \tag{177}$$

random times up to precision $\mathcal{O}(\epsilon/\,\mathrm{polylog}(\epsilon^{-1}))$, is sufficient to obtain with probability of success at least $2/3$ an estimate $\hat{a}_\alpha$ of a_α satisfying

$$|\hat{a}_\alpha - a_\alpha| = \epsilon. \tag{178}$$

Proof. By Eq. (175), measuring the expectation value of P under the time evolution will yield values that are within $\mathcal{O}(\epsilon/\log(\epsilon^{-1}))$ of the expectation value for the evolution under $\mathcal{L}$. As the polynomial interpolation method we use is robust to noise on the values of the polynomial and we only need the expectation value of local Paulis for our recovery, the statement follows. □

Thus, we see that, in general, as long as the perturbation to the idealized, local Lindbladian is of the same order as the estimate's precision required at each step for local Paulis, we can still recover the parameters of the idealized Lindbladian.

A. Robustness to perturbations

Our results strongly rely on the fact that local quantum dynamics satisfy LR bounds. Thus, it is natural to what extend our protocol is robust against global perturbations, i.e. what happens if we have small couplings between distant parts of the system. Let us now show that our protocol is robust against such perturbations.

To model such perturbations, we will assume that the true generator is of the form

$$\mathcal{L} = \mathcal{L}' + \Delta, \tag{179}$$

where $\mathcal{L}'$ is a time evolution that satisfies a LR bound and Δ is an arbitrary superoperator that is supposed to model the non-local parts of the evolution. Furthermore, we will define the following expansion quantity $\kappa(\Delta, k)$ that measures the impact of the non-local part on observables supported on at most k sites:

$$\kappa(\Delta, k) = \sup_{O:\mathrm{supp}(O)\le k, \|O\|\le 1} \|\Delta(O)\|. \tag{180}$$

To see why this quantity measures the impact of global perturbations on local observables, let us consider the case where we have

$$\Delta(X) = \tau \sum_{i,j=1, i\neq j}^{n} i[H_{i,j}, X], \tag{181}$$

for some arbitrary Hamiltonian evolution terms $\|H_{i,j}\| \leq 1$ supported on qubits i, j. That is, this additional term models the situation where we have a small interactions between all pairs of qubits. Note that the norm of this perturbation scales like $\sim n^2$ in general. In contrast, $\kappa(\Delta, k) \sim \tau k n$. This because for an observable supported on k sites, only the terms that also interact with that site will act nontrivially on it. So we have a total of $\sim nk$ terms acting on that site.

We then have:

Theorem 9.1. *Let $\mathcal{L}$ be a Lindbladian and suppose it is of the form*

$$\mathcal{L} = \mathcal{L}' + \Delta, \tag{182}$$

where $\mathcal{L}'$ satisfies a LR bound as in Eq. (142)*. Then we have for an observables O s.t. $\|O\| \leq 1$ and*

$$\|e^{t\mathcal{L}} - e^{t\mathcal{L}'}(O)\| \leq \inf_{\delta>0} t\delta\|\Delta\|_{+\infty\to+\infty} + \int_0^t ds\kappa(\Delta, |B(\mathrm{supp}(O), \log(\delta^{-1}) + vs))|). \tag{183}$$

Proof. We have that:

$$(e^{t\mathcal{L}} - e^{t\mathcal{L}'})(O) = \int_0^t ds e^{(t-s)\mathcal{L}}\Delta e^{s\mathcal{L}'}(O). \tag{184}$$

Now for any $\delta > 0$ we can approximate $e^{s\mathcal{L}'}(O)$ up to δ in operator norm by an observable supported on a ball of radius $r_{s,\delta} = vs + \log(\delta^{-1})$ around the original support of O, i.e.:

$$e^{s\mathcal{L}'}(O) = O_{r_s} + \delta\tilde{O}_s \tag{185}$$

where $\|O_{r_s}\|, \|\tilde{O}_s\| \leq 1$ and $\tilde{O}_s$ does not necessarily have any further locality. Now:

$$\|(e^{t\mathcal{L}} - e^{t\mathcal{L}'})(O)\| \leq \int_0^t ds\|e^{(t-s)\mathcal{L}}\Delta e^{s\mathcal{L}'}(O)\| \leq \int_0^t ds(\|e^{(t-s)\mathcal{L}}\Delta O_{r_{s,\delta}}\| + \|e^{(t-s)\mathcal{L}}\Delta(\delta\tilde{O}_s)\|). \tag{186}$$

We can estimate the first summand of the integral by

$$\int_0^t ds\|e^{(t-s)\mathcal{L}}\Delta(O_{r_{s,\delta}})\| \leq \int_0^t ds\kappa(\Delta, |B(\mathrm{supp}(O), \log(\delta^{-1}) + vs)|) \tag{187}$$

as, by definition, $\|\Delta(O_{r_{s,\delta}})\| \leq \kappa(\Delta, r(s,\delta))$ and $e^{(t-s)\mathcal{L}}$ is a quantum channel in the Heisenberg picture and cannot increase the operator norm. We can estimate the second summand in a similar way and get:

$$\int_0^t ds\|e^{(t-s)\mathcal{L}}\Delta(\delta\tilde{O}_s)\| \leq \delta t\|\Delta\|_{+\infty\to+\infty}. \tag{188}$$

As $\delta > 0$ here was arbitrary, we can further optimize the bound over all $\delta > 0$, which gives the claim. □

In a nutshell, the bound above says that our protocol is only sensitive to the extent at which global couplings affect local observables, which makes it significantly more robust than one would expect naively. Let us illustrate the scaling of the bound above with the example above of all-to-all couplings in Eq. (181). By a triangle inequality, $\|\Delta\| \leq \tau\frac{n(n-1)}{2}$. Thus, for an observable initially supported on a constant number of sites, we have that:

$$\kappa(\Delta, r(s,\delta)) = \tau n(vs + C\log(\delta^{-1}))^D, \tag{189}$$

From this we conclude that for such all-to-all perturbations, we have that

$$\|(e^{t\mathcal{L}} - e^{t\mathcal{L}'})(O)\| = \inf_{\delta>0} \mathcal{O}(n\tau(\delta tn + t^{D+1}\log^D(\delta^{-1}))) = \mathcal{O}(n\tau(t + t^{D+1}\log^D(n))), \tag{190}$$

where for the last bound we picked $\delta = n^{-1}$. Thus, we see that as long as $nt\tau \ll \epsilon$, our protocol still produces reliable results even under such global perturbations. Also note that our more refined perturbation bound using the LR-bound is quadratically better than the naive bound $n^2 t\tau \ll \epsilon$ we directly obtain from observing that $\|\Delta\|_{+\infty\to+\infty} \leq n^2$.

B. Robustness to characterized SPAM errors

Let us consider how to adapt our protocol to the case where we have SPAM errors. We will model such errors by assuming we know quantum channels T_P and T_M that model the noise when preparing the state and the measurement and we assume that these channels are independent of the Pauli basis we are measuring or preparing.

In that case, the process shadow tomography would be estimating the value of

$$2^{-n}\,\mathrm{tr}\,(P_a(T_M \circ e^{t\mathcal{L}} \circ T_P)(P_b)) = 2^{-n}\,\mathrm{tr}\,(T_M^*(P_a)e^{t\mathcal{L}}(T_P(P_b)). \tag{191}$$

If the observable $T_M^*(P_a)$ is sufficiently local, i.e. it does not increase the support of P_a signigicantly, then our previous results hold and we know that the function $f: t \mapsto 2^{-n}\,\mathrm{tr}\,(T_M^*(P_a))e^{t\mathcal{L}}(T_P(P_b))$ will be well-approximated by a polynomial of low-degree. Thus, we can see that we can evaluate the derivative of the function at 0 exactly as before. However, now will get access to the equation:

$$2^{-n}\,\mathrm{tr}\,(T_M^*(P_a))\mathcal{L}(T_P(P_b)) = c_{a,b} \tag{192}$$

If the channels T_M, T_P are sufficiently simple (i.e. approximately product), then it is possible to expand the expectation value in Eq. (192) efficiently and obtain a linear equation for the parameters of $\mathcal{L}$. Note, however, that these SPAM errors might decrease the condition number of the system of equations. Thus, it would be necessary to see how the condition number changes with the noise and then adjust the precision with which we estimate the derivative accordingly.

Let us exemplify this in a simple form of SPAM: local depolarizing noise. Let us assume that both T_M and T_P consist of $\mathcal{D}_p^{\otimes n}$, where for some parameter p $D_p(\rho) = p\rho + (1-p)\frac{I}{2}$. In this case, we see that $T_P(P_b) = p^{w(P_b)}P_b, T_M^*(P_a) = p^{w(P_a)}P_a$. Thus, in this case we see that the expectation values are rescaled by a factor $p^{w(P_a)+w(P_b)}$ and we can run exactly the same protocol, but now with a sampling overhead of p^{2k} to learn $k-$local Hamiltonians. In conclusion, we see that for noise models like local depolarizing (or in fact any Pauli noise), it is easy to recover our original results with a mild sampling overhead by adjusting the linear systems.

Developing an analytical understanding of how the condition number changes for other important noise models like amplitude damping is beyond the scope of this work. But we once again emphasize that this is straightforward and efficient to do numerically.

C. Robustness to unknown SPAM errors

In the last subsection we discussed how to deal with SPAM errors if they are well-characterized and independent of the measurement and state being prepared. Now we are going to discuss the case in which they are unknown and can potentially depend on the measurement and state we wish to prepare. We will show that the effect of SPAM errors will be local, in the sense that as long as the *local* SPAM rate is of order at most ϵ, then the protocol still yields reliable results. For simplicity, we will consider SPAM errors that are modelled by single-qubit product quantum channels. However, our proof works analogously for channels with an approximately finite light-cone. That is, they map local observables to observables that are approximately local. More formally, in the SPAM-free setting, our protocol entails measuring states of the form:

$$(\otimes_{i=1}^n V_i)e^{t\mathcal{L}}(\otimes_{i=1}^n U_i)(|0^n\rangle\langle 0^n|), \tag{193}$$

where U_i, V_i are single-qubit Pauli rotations. We then estimate local observables on such states. We will now model that instead we prepare the state

$$(\otimes_{i=1}^n T_{i,V_i})(\otimes_{i=1}^n V_i)e^{t\mathcal{L}}(\otimes_{i=1}^n U_i)(\otimes_{i=1}^n R_{i,V_i})(|0^n\rangle\langle 0^n|), \tag{194}$$

where T_{i,U_i} and R_{i,V_i} are single-qubit quantum channels that model the SPAM errors and can potentially depend on the gates we are implementing. We will now see that only local SPAM errors affect the performance of our protocol. But before that, let us discuss the sensitivity of the shadows protocol of Sec. 8 to noise. Suppose we wish to use the shadow protocol to estimate a pair of Paulis P_a, P_b of respective weight w_a, w_b, i.e. we wish to estimate

$$2^{-n}\,\mathrm{tr}\left[P_b e^{t\mathcal{L}}(P_a)\right]. \tag{195}$$

Then the protocol discussed in Sec. 8 consists of measuring an observable of $O_{a,b,\{V_i\},\{U_i\}}$ that depends on the state preparation and measurement unitaries, is supported only on the support of P_b and such that $\|O_{a,b,\{V_i\},\{U_i\}}\| = 3^{w_a+w_b}/4$ over an ensemble of random states. Denoting by B the support of P_b, we conclude that if for all possible U_i, V_i we have

$$\|\,\mathrm{tr}_{B^c}\left[(\otimes_{i=1}^n T_{i,V_i})(\otimes_{i=1}^n V_i)e^{t\mathcal{L}}(\otimes_{i=1}^n U_i)(\otimes_{i=1}^n R_{i,V_i}) - (\otimes_{i=1}^n V_i)e^{t\mathcal{L}}(\otimes_{i=1}^n U_i)(|0^n\rangle\langle 0^n|)\right]\|_1 \leq \tau, \tag{196}$$

then by an application of Hölder's inequality, we have that the expectation value of the shadows protocol will deviate by at most $\tau 3^{w_a+w_b}/4$ from the ideal value. Although this exponential scaling of the sensitivy in the locality is undesirable, if the SPAM errors are very strong, we can also adopt the less efficient strategy of preparing each state for the isolation separately. As for that case we only need to measure a local observable of operator norm 1, we see that we avoid the exponential factor and the overall error in the estimation for the observable directly follows from Eq. (196) and corresponds to τ.

Let us now show that τ does not scale with the system's size and only depends on the local noise rate:

Proposition 9.1. *In the notations above, assume that for all i, U_i, V_i we have that $\|R_{i,V_i} - id\|_\diamond, \|T_{i,V_i} - id\|_\diamond \leq \tau$. Furthermore, assume that $\mathcal{L}$ satisfies a LR-bound like that of Eq. (81). Then for $t, w_a, w_b = \mathcal{O}(1)$ we have that:*

$$\|\,\mathrm{tr}_{B^c}\left[(\otimes_{i=1}^n T_{i,V_i})(\otimes_{i=1}^n V_i)e^{t\mathcal{L}}(\otimes_{i=1}^n U_i)(\otimes_{i=1}^n R_{i,V_i}) - (\otimes_{i=1}^n V_i)e^{t\mathcal{L}}(\otimes_{i=1}^n U_i)(|0^n\rangle\langle 0^n|)\right]\|_1 = \mathcal{O}(\tau\mathrm{polylog}(\tau^{-1})). \tag{197}$$

In particular, under such a SPAM error we have that the process shadow protocol for Paulis P_a, P_b will have an expectation value that is $\mathcal{O}(3^{w_a+w_b}\mathrm{poly}(w_a, w_b)\tau\mathrm{polylog}(\tau^{-1}))$ close to the SPAM-free setting.

Proof. the statement is equivalent to having for all O_B supported on B s.t. $\|O\| \leq 1$ that

$$\mathrm{tr}\left[O_B\left[\otimes_{i=1}^n T_{i,V_i})(\otimes_{i=1}^n V_i)e^{t\mathcal{L}}(\otimes_{i=1}^n U_i)(\otimes_{i=1}^n R_{i,V_i}) - (\otimes_{i=1}^n V_i)e^{t\mathcal{L}}(\otimes_{i=1}^n U_i)(|0^n\rangle\langle 0^n|)\right]\right] = \mathcal{O}(\tau\mathrm{polylog}(\tau^{-1})). \tag{198}$$

First, note that we have that

$$\mathrm{tr}\left[O_B(\otimes_{i=1}^n T_{i,V_i})(\otimes_{i=1}^n V_i)e^{t\mathcal{L}}(\otimes_{i=1}^n U_i)(\otimes_{i=1}^n R_{i,V_i})(|0^n\rangle\langle 0^n|)\right] = \tag{199}$$

$$\mathrm{tr}\left[O_B(\otimes_{i\in B} T_{i,V_i})(\otimes_{i\in B} V_i)e^{t\mathcal{L}}(\otimes_{i=1}^n U_i)(\otimes_{i=1}^n R_{i,V_i})(|0^n\rangle\langle 0^n|)\right] = \tag{200}$$

$$\mathrm{tr}\left[O'_B e^{t\mathcal{L}}(\otimes_{i=1}^n U_i)(\otimes_{i=1}^n R_{i,V_i})(|0^n\rangle\langle 0^n|)\right] + \epsilon_1, \tag{201}$$

where $|\epsilon| \leq w_b\tau$ and O'_B is again an observable of operator norm at most 1 on B. Here we have used that all the unitaries and channels acting outside of B will have a trivial effect. For those acting on B we use our bound on the diamond distance to the identity channel. Now, by our LR bound we have that for times $t = \mathcal{O}(1)$, we can write $e^{t\mathcal{L}^*}(O'_B) = O_{t,B(t,r)} + X_{r,t}$, where $O_{t,B(t,r)}$ is an observable supported on a ball of size $tv + r$ around B and $\|X_{r,t}\| \leq \mathcal{O}(e{-}Cr)$. In particular, picking $r = \mathcal{O}(\log(\tau^{-1}))$ and as $t = \mathcal{O}(1)$ we get that $e^{t\mathcal{L}^*}(O'_B)$ is localized on a number of sites that is at most $w_b(vt + C\log(\tau^{-1}))^D$ up to an error ϵ, where D is the dimension of the lattice.

Thus:

$$\mathrm{tr}\left[O'_B e^{t\mathcal{L}}(\otimes_{i=1}^n U_i)(\otimes_{i=1}^n R_{i,V_i})(|0^n\rangle\langle 0^n|)\right] = \mathrm{tr}\left[O'_{t,B(t,r)}(\otimes_{i=1}^n U_i)(\otimes_{i=1}^n R_{i,V_i})(|0^n\rangle\langle 0^n|)\right] + \epsilon_2, \tag{202}$$

where $\epsilon_2 \leq \tau$ by our choice of r. We can then follow the same procedure as before for the T_{i,V_i} to conclude that only those channels on the support of $O'_{t,B(t,r)}$ contribute to the noise. Thus, we get an additional error of order $\mathcal{O}(\tau\mathrm{polylog}(\tau^{-1}))$. This concludes the proof. □

Recall that for the protocols discussed here we have $w(P_a), w(P_b) = \mathcal{O}(1)$. Thus, we see that our protocol is robust to local, gate-dependent SPAM noise as long as the *local* noise rates are smaller than the target accuracy and the SPAM errors do not need to decrease with the system's size.

Supplementary Note 10: Learning the structure of the interactions

In the main text we assumed that we know which qubits interact with which qubits, i.e. the interaction graph is known, and we only wish to recover the parameters of this interaction. However, a close inspection of the proof shows that we do not really require exact knowledge of the structure; all we need is the promise that the evolution satisfies a LR bound. Indeed, the only structure required for our proofs to go through is that the evolution of local observables can be well-approximated by that of truncated Lindbladian, and this remains true if the structure of the interactions is unknown, but the true evolution still satisfies a LR bound.

Thus, in this section we will discuss how to apply our protocol in the setting in which we expect the evolution to satisfy a LR bound, but we do not necessarily know all qubits that are coupled to each other. Discussing this setting in full detail and generality goes beyond the scope of this paper, so we will restrict to the case where there is no Lindblad term and we restrict to interaction acting on only two qubits.

Furthermore, we will make the assumption that we have a lower-bound on the strength of all the interactions that are non-zero. This is necessary to obtain bounds on the performance of the protocol, as if we don't have such a lower-bound it will in principle be necessary to measure expectation values with a very high precision to see their effect.

We will thus assume that our generator is of the form

$$\mathcal{L}(X) = \sum_{(i,j)\in E} a_\alpha i[H_\alpha, X], \tag{203}$$

where the (unknown) interaction graph $G = (V, E)$ is such that the evolution induced by $\mathcal{L}$ satisfies a LR bound as in Eq. (81), $1 \geq |a_\alpha| \geq \eta$ and $\|H_\alpha\| \leq 1$ and H_α is supported on at most 2 qubits.

Proposition 10.1 (Learning the interaction graph)**.** *Let $\mathcal{L}$ be a Lindbladian on n-qubits as described in Eq.* (203) *that satisfies a LR bound as in Eq.* (81) *and $1 \geq |a_\alpha| \geq \eta$. Suppose we can measure the expectation value of two-body Pauli observables in the time interval $[t_0, t_{\max}]$ under $\{e^{t\mathcal{L}}\}_{t_0\leq t\leq t_{\max}}$ for*

$$t_0^{-1} = \mathcal{O}\left[\mathrm{polylog}(\eta^{-1})\right] \tag{204}$$

and $t_{\max} = 2 + t_0$. Then, measuring the evolution in Pauli bases on Pauli eigenstates under the evolution of $e^{t\mathcal{L}}$ for

$$m = \mathcal{O}\Big[\mathrm{polylog}(\epsilon^{-1})\Big] \tag{205}$$

random times $\mathcal{O}(\eta^{-2}\log(n)\,\mathrm{polylog}(\eta^{-1}))$ times each, is sufficient to obtain with probability of success at least $2/3$ the list of the edges E.

Proof. The protocol is as follows: we first perform the process shadow tomography protocol for m random times t_i and measure the output $\mathcal{O}(\eta^{-2}\log(n)/\,\mathrm{polylog}(\eta^{-1}))$ times for each t_i. Then, by a union bound, we will obtain the expectation value of all the $\mathcal{O}(n^4)$ pairs input and output 2-body Pauli strings up to precision $\eta/\,\mathrm{polylog}(\eta^{-1})$ each with probability at least 2/3. Conditioning on the event that all of the estimates were correct, we can use our previous results to estimate the interaction strength for all 2−body Pauli terms up to precision $\eta/4$. We then set an edge between all qubits such that they have a 2−body interaction at least $\frac{3}{4}\eta$ in absolute value. By our assumption that all the interactions are at least η, we will then identify all interactions correctly. □

Thus, we see that our protocol can also correctly identify the interactions as long as some LR-bound is given. To the best of our knowledge, this is the first protocol that does not necessarily require knowledge of the graph, just some assumptions on the locality. Furthermore, note that the sample complexity of this version of the protocol requires approximately the same number of samples as to estimate the values themselves. However, note that learning the graph is computationally more expensive. This is because we need to run $\mathcal{O}(n^2)$ polynomial interpolations to estimate all couplings, whereas when we are given the graph, the number of interpolation steps required is $\mathcal{O}(n)$.

---

[1] W. Zhang, N. Konstantinidis, K.A. Al-Hassanieh, and V.V. Dobrovitski. Modelling decoherence in quantum spin systems. *Journal of Physics: Condensed Matter*, 19(8):083202, 2007.

[2] V.V. Dobrovitski and H.A. De Raedt. Efficient scheme for numerical simulations of the spin-bath decoherence. *Physical Review E*, 67(5):056702, 2003.

[3] M. Ledoux. *The concentration of measure phenomenon.* Number 89. American Mathematical Soc., 2001.

[4] C.P. Slichter. *Principles of magnetic resonance*, volume 1. Springer Science & Business Media, 2013.

[5] R. Kubo, M. Toda, and N. Hashitsume. *Statistical physics II: nonequilibrium statistical mechanics*, volume 31. Springer Science & Business Media, 2012.

[6] J. Dalibard, Y. Castin, and K. Mølmer. Wave-function approach to dissipative processes in quantum optics. *Physical review letters*, 68(5):580, 1992.

[7] M.A. Nielsen and I. Chuang. Quantum computation and quantum information, 2002.

[8] M. B. Hastings. Locality in Quantum Systems. *arXiv:1008. 5137 [math-ph, physics:quant-ph]*, August 2010. arXiv: 1008. 5137.

[9] D. Poulin. Lieb-Robinson Bound and Locality for General Markovian Quantum Dynamics. *Phys. Rev. Let.*, 104(19):190401, May 2010.

[10] T. Barthel and M. Kliesch. Quasilocality and efficient simulation of markovian quantum dynamics. *Phys. Rev. Let.*, 108(23):230504, 2012.

[11] M. Kliesch, C. Gogolin, and J. Eisert. Lieb-Robinson Bounds and the Simulation of Time-Evolution of Local Observables in Lattice Systems. In Volker Bach and Luigi Delle Site, editors, *Many-Electron Approaches in Physics, Chemistry and Mathematics*, pages 301–318. Springer International Publishing, Cham, 2014. Series Title: Mathematical Physics Studies.

[12] D. Kane, S. Karmalkar, and E. Price. Robust Polynomial Regression up to the Information Theoretic Limit. In *2017 IEEE 58th Annual Symposium on Foundations of Computer Science (FOCS)*, pages 391–402, Berkeley, CA, October 2017. IEEE.

[13] W. Markoff and J. Grossmann. über Polynome, die in einem gegebenen Intervalle möglichst wenig von Null abweichen. *Mathematische Annalen*, 77(2):213–258, June 1916.

[14] T.M. Rassias and V. Gupta, editors. *Mathematical Analysis, Approximation Theory and Their Applications*. Number 111 in Springer Optimization and Its Applications. Springer International Publishing : Imprint: Springer, Cham, 1st ed. 2016 edition, 2016.

[15] J. Haah, R. Kothari, and E. Tang. Optimal learning of quantum Hamiltonians from high-temperature Gibbs states, 2021. arXiv:2108. 04842v1.

[16] E. H. Lieb and D. W. Robinson. The finite group velocity of quantum spin systems. *Commun. Math. Phys*, 28(3):251–257, sep 1972.

[17] M. Kliesch, C. Gogolin, and J. Eisert. Lieb-robinson bounds and the simulation of time-evolution of local observables in lattice systems. In *Many-Electron Approaches in Physics, Chemistry and Mathematics*, pages 301–318. Springer International Publishing, 2014.

[18] R. Sweke, J. Eisert, and M. Kastner. Lieb–robinson bounds for open quantum systems with long-ranged interactions. *Journal of Physics A: Mathematical and Theoretical*, 52(42):424003, 2019.

[19] T. S. Cubitt, A. Lucia, S. Michalakis, and D. Perez-Garcia. Stability of local quantum dissipative systems. *Commun. Math. Phys*, 337(3):1275–1315, 2015.

[20] T. Kuwahara and K. Saito. Strictly Linear Light Cones in Long-Range Interacting Systems of Arbitrary Dimensions. *Phys. Rev. X*, 10(3):031010, July 2020.

[21] B. Nachtergaele, A. Vershynina, and V. A. Zagrebnov. Lieb-robinson bounds and existence of the thermodynamic limit for a class of irreversible quantum dynamics. *AMS Contemporary Mathematics*, 552:161–175, 2011.

[22] Zhiyuan Wang and Kaden RA Hazzard. Tightening the lieb-robinson bound in locally interacting systems. *PRX Quantum*, 1(1):010303, 2020.

[23] J. Kunjummen, M.C. Tran, D. Carney, and J.M. Taylor. Shadow process tomography of quantum channels, 2021. arXiv:2110.03629v2.

[24] R. Levy, D. Luo, and B.K. Clark. Classical Shadows for Quantum Process Tomography on Near-term Quantum Computers, 2021. arXiv:2110.02965v1.

[25] L. Devroye, M. Lerasle, G. Lugosi, and R.I. Oliveira. Sub-gaussian mean estimators. *The Annals of Statistics*, 44(6):2695–2725, 2016.
